# Insights into Endophytic and Rhizospheric Bacteria of Five Sugar Beet Hybrids in Terms of Their Diversity, Plant-Growth Promoting, and Biocontrol Properties

**DOI:** 10.1007/s00248-023-02329-0

**Published:** 2023-12-27

**Authors:** Marija Petrović, Tamara Janakiev, Milica Ljaljević Grbić, Nikola Unković, Tatjana Stević, Slavoljub Vukićević, Ivica Dimkić

**Affiliations:** 1 Faculty of Biology, University of Belgrade Studentski trg 16, Belgrade, 11158 Serbia; 2 Institute for Medicinal Plant Research “Dr Josif Pančić,” Tadeuša Košćuška 1, Belgrade, 11000 Serbia; 3 FERTICO DOO, Vojvode Putnika 94a, 22320, Indjija, Serbia

**Keywords:** Plant-associated bacteria, Biotic and abiotic stresses, Sugar beet hybrids, *Cercospora-*resistant hybrid, Keystone species

## Abstract

Sugar beet is the most important crop for sugar production in temperate zones. The plant microbiome is considered an important factor in crop productivity and health. Here, we investigated the bacterial diversity of seeds, roots, and rhizosphere of five sugar beet hybrids named Eduarda (ED), Koala (KO), Tibor (T), Tajfun (TF), and *Cercospora*-resistant (C). A culture-independent next-generation sequencing approach was used for the further investigation of seed-borne endophytes. Hybrid-associated bacteria were evaluated for their plant growth–promoting (PGP) characteristics, antagonistic activity towards *Cercospora beticola* and several *Fusarium* strains in dual culture assays, and drought and salinity tolerance. High-throughput sequencing revealed that the *Proteobacteria* phylum was most dominant in the seeds of all hybrids, followed by *Cyanobacteria* and *Actinobacteriota*. The predominant genus in all hybrids was *Pantoea*, followed by *Pseudomonas*, *Acinetobacter*, *Chalicogloea*, *Corynebacterium*, *Enterobacter*, *Enterococcus*, *Glutamicibacter*, *Kosakonia*, and *Marinilactibacillus*. Unique genera in the hybrids were *Pleurocapsa* and *Arthrobacter* (T), *Klebsiella* (TF), *Apibacter* (ED), and *Alloscardovia* (KO). The genera that were most represented in one hybrid were *Weissella* and *Staphylococcus* (TF); *Streptococcus* (T); *Gardnerella*, *Prevotella*, and *Rothia* (KO); and *Gilliamella*, *Lactobacillus*, and *Snodgrassella* (ED). Thirty-two bacteria out of 156 isolates from the rhizosphere, roots, and seeds were selected with respect to various plant growth–promoting activities *in vitro*, i.e., nitrogen fixation, phosphate solubilization, siderophore production, indole-3-acetic acid production, 1-aminocyclopropane-1-carboxylic acid deaminase activity, hydrogen cyanide production, exoenzymatic activity (amylase, protease, lipase, cellulase, xylanase, mannanases, gelatinase, and pectinase), mitigation of environmental stresses, and antifungal activity. *Mixta theicola* KO3-44, *Providencia vermicola* ED3-10, *Curtobacterium pusillum* ED2-6, and *Bacillus subtilis* KO3-18 had the highest potential to promote plant growth due to their multiple abilities (nitrogen fixation, phosphate solubilization, production of siderophores, and IAA). The best antagonistic activity towards phytopathogenic fungi was found for *Bacillus velezensis* C3-19, *Paenibacillus polymyxa* C3-36 and *Bacillus halotolerans* C3-16/2.1. Only four isolates *B. velezensis* T2-23, *B. subtilis* T3-4, *B. velezensis* ED2-2, and *Bacillus halotolerans* C3-16/2.1 all showed enzymatic activity, with the exception of xylanase production. *B. halotolerans* C3-16/2.1 exhibited the greatest tolerance to salinity, while two *B. subtilis* strains (C3-62 and TF2-1) grew successfully at the maximum concentration of PEG. The current study demonstrates that sugar beet–associated bacteria have a wide range of beneficial traits and are therefore highly promising for the formulation of biological control and PGP agents.

**Supplementary Information:**

The online version contains supplementary material available at 10.1007/s00248-023-02329-0.

## Introduction

Plants live in a close symbiotic relationship with their microbiota and are thus regarded as holobionts where the cells of the plant host are typically outnumbered by the related microorganisms [1, 2]. Depending on the plant environment, plant-associated microbiota can be found on the exterior of plants, such as the rhizosphere or phyllosphere, or in the interior of plants, referred to as the endosphere [2]. The soil harbors a wide variety of microorganisms, with the root microbiota mainly acquired from the soil through horizontal transfer. Additionally, seeds serve as another crucial source of microorganisms, vertically transmitting their microbiota, and some bacteria can thrive in the roots of growing plants [3]. The presence of particular co-occurrence patterns and microbial networks highlights the fact that the colonization of plants by microbes is a targeted process [2]. Distinct plant species grew in symbiosis with remarkably different microbial communities in the rhizosphere and root compartments while occupying the same soil environment [3]. The structure of plant roots and their exudates, such as amino acids, phenolic compounds, organic acids, sugars, and other small secondary metabolites, plays an important role in shaping the rhizosphere microbiome. Variations in the plant genome affect the composition of exudates and root structure, resulting in different microbiome assemblies. It has been reported that even a minor genetic variation of the host plant can affect its microbiome composition [4]. The composition of plant secondary metabolites is also influenced by their associated microbiota which results in the development of new metabotypes as has been shown recently [2]. Some plants under the phosphate or iron deficiency tend to increase the secretion of citrate, malate, or oxalate to enrich the rhizosphere with organic carbon, which attracts beneficial microorganisms [5]. While previous research was focused more on the plant-pathogen relationship, discoveries from the past decade elucidate significance of the beneficial interaction between microbes and their hosts. Strong involvement of microbial diversity in the development of these interactions was recognized [6]. Plant growth–promoting bacteria (PGPB) are beneficial bacteria that can stimulate plant growth through various mechanisms such as nitrogen fixation, phosphate solubilization, production of siderophores, indole-3-acetic acid (IAA), and 1-aminocyclopropane-1-carboxylic acid (ACC) deaminase [7]. Furthermore, PGPBs are recognized for the protection of host plants against phytopathogens via different mechanisms that involve the production of antimicrobial components, hydrolytic enzymes, competition for nutrients, and induction of resistance in the host plant [8]. Drought and high salinities are one of the limiting factors in the growth and productivity of agricultural crops, especially in arid and semi-arid regions. As a natural hazard, drought in Serbia in the summer of 2017 severely affected agricultural production, reducing yields of corn, sunflower, soybeans, and sugar beets between 30 and 60%, while the total loss was estimated at $1.5 billion [9]. In this regard, it has been reported that PGPBs enhanced tolerance to drought and salt stress of many different crops [10]. Moreover, recent research has revealed that the plant microbiome also contributes to cold acclimation, a key element restricting plant geographic range as well as crop development and yield in specific regions [2]. Thus, the plant microbiome is acknowledged as a significant driver of plant productivity and health.

The population of the world is anticipated to increase steadily and will total 9.8 billion in 2050 and 11.2 billion in 2100. Simultaneously, there will be less area accessible for crop production, indicating that agricultural management and technologies must be used to enable intensification of crop production [11]. A more sustainable approach to farming may result from manipulating the plant microbiome, which also has the potential to boost agricultural output, decrease chemical inputs, and lower greenhouse gas emissions. This goal is considered essential for supporting the world’s expanding population [12].

Sugar beet is the most important crop for sugar production in temperate zones, while it is second in the world, covering 30% of the world sugar production. Recently, sugar beet has also been recognized as a source for biofuel production [13, 14]. Unfortunately, sugar beet production is exposed to several phytosanitary problems which include various fungal pathogens resulting in significant losses in crop yield and quality [15]. *Cercospora* leaf spot is one of the most widespread and destructive foliar diseases of sugarbeet caused by *Cercospora beticola* [15]. Seeds, seedlings, roots, and leaves are affected by *Fusarium* species, among which *F. oxysporum* causes various diseases including *Fusarium* yellow, seedling wilt, and root rot [16]. *Fusarium* wilt was also recorded for been, tomato, and cowpea, whereas *F. equiseti* poses a serious threat for significant yield losses [17]. *Fusarium solani* is a causal agent of *Fusarium* root rot of sugar beets in the USA [18]. Recently, several *Fusarium* species were isolated from sugar beet roots that showed symptoms of root rot, including *F. graminearum*, *F. nygamai*, *F. equiseti*, and *F. verticillioides* [18]. To date, they have not been characterized as sugar beet pathogens, but in the pathogenicity test, all listed species caused root rot symptoms similar to natural infections [18]. The use of fungicides has been effective in controlling these diseases under field conditions [15]. However, the use of fungicides for crop protection is associated with several negative consequences, such as the emergence of resistant strains of pathogens, the reduction of the number of beneficial microorganisms in the microbiome, and the accumulation of toxic substances in the soil [19]. The use of microorganisms such as bacteria to control fungal diseases and stimulate crop growth is a strategy that is part of sustainable and eco-friendly production.

**Fig. 1:**
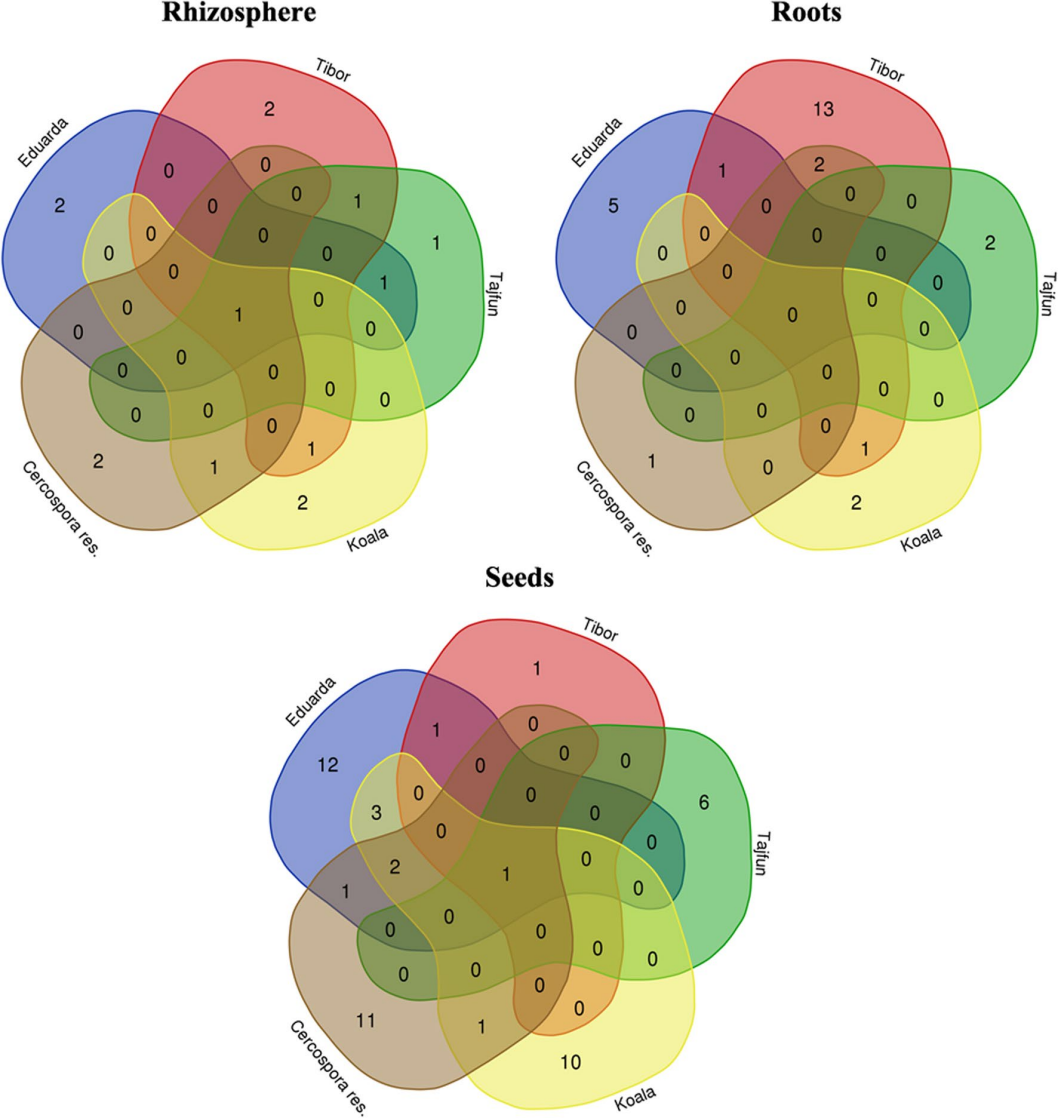
Venn diagram showing the common and unique distribution of culturable bacterial species in the rhizosphere, roots, and seeds among five different sugar beet hybrids

Exploring plant-microbe interactions and community building provides vital insights into plants as metaorganisms. This holistic understanding enhances our knowledge of mutual benefits between plants and microbes, leading to sustainable agriculture and improved crop resilience in challenging environments. With more information about the plant microbiota in relation to biotic and abiotic stresses, plant genotypes, and environmental conditions, it may be possible to identify more suitable candidates or approaches for inoculation in a given environment. The microbiome of the rhizosphere, roots, and seeds of various sugar beet cultivars has been reported [20–22]. We have closely examined the bacterial diversity in the rhizosphere, roots, and seeds of the most commonly used sugar beet hybrids (Eduarda, Koala, Tibor, Tajfun, and the *Cercospora*-resistant hybrids) in Serbia (Fig. 1). In addition, our study represents a pioneering investigation of the diversity of total seed-borne endophytic bacteria using the next-generation sequencing (NGS) approach. By culturing endophytic (roots and seed) and rhizospheric bacteria and screening them for plant growth–promoting properties, enzyme production, stress tolerance, and antifungal potential, we also intend to identify important microbial strains with beneficial properties. Our results should contribute to the development of targeted agricultural strategies by providing insights into specific microbial traits that can improve sugar beet health and overall crop performance.

## Materials and Methods

### Plant Material

Five hybrids of sugar beet, i.e., Eduarda, Koala, Tibor, Tajfun, and *Cercospora*-resistant, were used in this study, hereafter referred as ED, KO, T, TF, and C, respectively. Plant samples and their surrounding rhizosphere of sugar beet hybrids were collected in May 2021 at the experimental field of Sunoko company in Kovačica, Serbia (45°05′06.7″N 20°38′07.2″E) at V2.1 growth stage (two leaves are unrolled, and the third leaf is just visible) [23] and transported to the laboratory in bags.

### *DNA Extraction*, *Library Preparation*, *and NGS*

Sugar beet seeds were obtained commercially by the Sunoko Research and Development Center. The seeds (10 seeds per hybrid) were sterilized with 70% ethanol, shaken for 1–2 min and then washed with water. The procedure was repeated with 3% sodium hypochlorite, and the samples were washed with water several times. After surface sterilization, the seed samples were crushed in a mortar. Extraction of total DNA was performed using a Quick-DNA^TM^ Faecal/Soil Microbe Miniprep Kit (Zymo Research, Irvine, CA, USA) according to the manufacturer’s instructions. The DNA was quantified using Qubit fluorometric quantitation (Qubit 4 fluorometer, Invitrogen™, Waltham, MA, USA). DNA samples were commercially sequenced by Novogene Co., Ltd. (Cambridge, UK) using a 2 × 250-bp paired-end run on a MiSeq Sequencer, according to the manufacturer’s instructions (Illumina, San Diego, CA, USA). The 16S rRNA gene-specific sequences to target the V3 and V4 regions (470 bp) were used in this study, with 341F (5′-CCTACGGGNGGCWGCAG-3′) and 805R (5′-GACTACHVGGGTATCTAATCC-3′) primers [24]

### Sequence Data Processing and Taxonomy Annotation

Following the initial quality check, primers were eliminated using cutadapt 3.4 in paired-end mode with default settings. Only read pairs containing both primers were subjected to further processing. Subsequently, sequence inference was performed using the dada2 R package [25]. Quality trimming of reads was accomplished using default parameters in the filterAndTrim function, with the additional criteria of right trimming after 223 nt for forward and reverse reads, and discarding reads shorter than 150 nt. Sequences with more than three expected errors for the forward and reverse strands, calculated using the quality score (*Q*), were discarded (maxEE = *c*(3, 3), where *Q* is the quality score represented as sum(10^(−*Q*/10))). To join paired sequences, a minimum overlap of 12 nt (default) was used. Chimera removal was performed with default options in removeBimeraDenovo. Taxonomy assignment up to the genus level was accomplished using the SILVA 138.1 (silva_nr99_v138.1) strain set (https://zenodo.org/record/4587955) with IDTAXA [26] using default parameters and a confidence threshold set to 50. After annotating using Silva 138.1 as described above, the taxonomy was further supplemented by conducting a blast search against the NCBI 16S ribosomal RNA database (accessed at May 5, 2022). Blast best hits (BBH) with at least 98% identical matches and at least 99% query coverage were used to annotate missing taxonomies, but only if the entire taxonomy between the BBH and Silva annotation, up to the highest annotated taxonomic level, matched. Species-level annotation was performed by exact sequence matching (0 mismatches) against the combined Silva species (silva_species_assignment_v138.1 at https://zenodo.org/record/4587955) and NCBI 16S ribosomal RNA databases (accessed at May 5, 2022). In cases where the amplicon sequence variants (ASVs) matched multiple sequences, a concatenated string of all exactly matched species was employed as the species-level annotation.

### Bioinformatic and Statistical Analyses

Alpha diversity, assessed at ASV, species, genus, family, and phylum levels using the phyloseq R package [27], was determined after rarefaction to a consistent depth (101,470 reads, corresponding to the sample with the lowest reads). Chao, ACE, OBS, Simpson, and Shannon indices were used for evaluation. Kruskal–Wallis one-way analysis of variance compared alpha diversity among the five hybrids, with Dunn’s test presenting significant differences denoted by distinct letters above the bars. Beta diversity, evaluated at ASV, species, and genus levels, utilized double principal coordinate analysis (DPCoA) [28]. Prior to ordination, singleton ASVs and those with a read sum below 30 across all samples were removed. Multivariate homogeneity of group dispersions was assessed via PERMDISP2 [29] on the DPCoA distance matrix. PERMANOVA (ADONIS) [30] tested compositional similarities among groups, and pairwise ADONIS analyses examined dissimilarities between groups. Differential abundance analysis, aggregated up to the species level following the same ordination as for DPCoA, was performed with the microbiomeMarker package [31] using DESeq2 [32], which employed default parameters. The Bonferroni method adjusted *p*-values, considering *p* < 0.01 as statistically significant. Differential abundance, aggregated up to the species level, excluded species present in only one sample or with a read sum below 30 across all samples before analysis.

### Isolation of Endophytic and Rhizospheric Bacteria from Sugar Beet Hybrids

Samples of the lower part of the roots, approx. 100 mg (five roots of one hybrid plant) and seeds (five seeds of each hybrid), were washed, cleaned of soil particles with tap water, and sterilized as described above. After surface sterilization, the root samples were dried and chopped into a previously sterilized mortar with sterile tweezers and scissors. The roots and seeds were then crushed in the mortar and transferred to the 1.5 mL of Luria–Bertani (LB) medium supplemented with glycerol (final concentration 20%). The prepared stocks were stored at −80 °C until the further use. Eight different nitrogen-free media (NFb, LGI, JNFb, LGI-P, modified LGI with acids, JMV, M-medium, and M-medium without biotin) were used to isolate culturable endophytic bacteria from seeds and roots, as summarized in the work of Baldani et al. [33]. Rich culture media such as tryptic soy agar (TSA), nutrient agar (NA), and glucose-yeast calcium carbonate (GYC) were also used. Of the starting material obtained from the seed sample, 100 μL was spread onto each isolation medium, while the root material was diluted 1:10 before application to the media and then incubated at 30 °C for 10 days. After the incubation period, morphologically distinct colonies were restreaked onto NA and incubated under the same conditions to obtain axenic cultures. For selective isolation of *Bacillus* species from the rhizosphere, the thermal inactivation method was used. In short, the soil sample of each hybrid was transferred to test tubes with 3 mL of nutrient broth medium (NB) and incubated at 80 °C for 10 min. Furthermore, the tubes were incubated at 30 °C until growth appeared. Cultures were streaked on the Luria–Bertani agar plates (LA), and colonies of different morphologies were further selected. Pure cultures of the selected bacterial isolates from all origins were stored in LB glycerol stocks at −80 °C until further use.

### Molecular Identification of Endophytic and Rhizospheric Bacteria from Sugar Beet Hybrids

Genomic DNA obtained from a bacterial colony was used for PCR amplification [34]. From pure cultures growing on NA plates, a single colony was picked with the top of the pipette tips and resuspended in 25 μL of sterile deionized water. The prepared samples were incubated at 98 °C for 10 min. Colony PCR amplifications were performed in a 25-μL reaction mixture containing 12.5 μL FastGene Taq ready mix with dye (NIPPON Genetics Europe, GmbH, Düren, Germany), 0.5 μL of each primer (10 μM), 5 μL of DNA template, and PCR water to final volume. Primers used for the amplification of 16S rRNA gene (1500 bp) were the following: fD1Funi-16SF (5′-AGAGTTTGATCCTGGCTCAG-3′) and Rp2Runi-16SR (5′-ACGGCTACCTTGTTAGGACTT-3′). The following cycling conditions were used: one step of initial denaturation at 95 °C for 5 min followed by 30 cycles of denaturation at 95 °C for 40 s, annealing at 54 °C for 40 s and extension at 72 °C for 90 s and final extension step at 72 °C for 7 min. All PCR amplicons were purified using a purification kit (Euroclone spinNAker Gel&PCR DNA Purification Kit, Italy) according to the manufacturer’s instructions, and later sequenced by Eurofins Genomics Europe Sequencing service (Wien, Austria) using the 907R-16S primer (5′-CCGTCAATTCMTTTRAGTTT-3′). The obtained sequences were searched for sequence homologs of the reference strains in the NCBI GenBank database using the BLASTn algorithm. Sequences of the most closely related strains were used to ensure taxonomic identification. Sequences were aligned using the CLUSTAL W algorithm implemented in the BioEdit 7.2.5 program, and nucleotide position was checked manually.

### Evaluation of Endophytic and Rhizospheric Sugar Beet Bacteria for Their Plant Growth-Promoting (PGP) Properties *In Vitro*

Unless otherwise stated, 5 μL of the overnight culture was used in all tests. The ability of the isolates to fix nitrogen was determined by subculturing on nutrient media with and without NH_4_Cl as a source of nitrogen [35]. Screening of phosphate solubilization was performed on the NBRIP agar medium according to the method of Nautiyal [36]. The potential of sugar beet isolates to produce indole-3-acetic acid (IAA) was evaluated by the modification of the method described by Gordon and Weber [37]. A full loop (1 μL size) of bacterial culture was inoculated into the 1 mL of IAA detection medium and shaken for 24 h at 30 °C. Medium without bacteria was used as a negative control. After incubation, tubes were centrifuged at 10,000 rpm for 10 min. Subsequently, 1 mL of supernatant was transferred to the new tube and mixed with 1 mL of Salkowski reagent. Prepared samples were incubated for 2 h and then evaluated based on the color. Depending on the intensity of the color change (light orange, dark orange and red), IAA production was scored as follows: low (+), medium (++), and high (+++). Siderophore production was characterized by the modification of the O-CAS method described by Pérez-Miranda et al. [38]. Of bacterial overnight culture, 10 μL was poured into sterile wells previously placed on NA plates and incubated at 30 °C for 24 h. After incubation, 10 mL of CAS medium was poured on each plate. Following CAS medium solidification, the wells were removed and the plates were incubated at 30 °C for 24 h. The appearance of clear zones around bacterial colonies was a confirmation of siderophore production. Qualitative assessment of 1-aminocyclopropane-1-carboxylic acid (ACC) deaminase activity was conducted following the methodology outlined by Gupta et al. [39]. Exopolysaccharide (EPS) production was determined by subculturing isolates on yeast mannitol agar (YMA) plates, using the streak-plate procedure [40]. The motility assay was performed according to the method of Kim and Surette [41]. The ability of the isolates to produce hydrogen cyanide (HCN) was determined using Cyantesmo indicator strips (Machery-Nagel GmbH&Co., Germany) as described by Knežević et al. [42].

### Evaluation of Endophytic and Rhizospheric Sugar Beet Bacteria for Extracellular Enzyme Production *In Vitro*

The ability of the isolates to produce extracellular enzymes was assessed by inoculating 5 μL of the overnight bacterial culture onto the specific solid media. After 24 or 48 h of incubation at 30 °C, the enzymatic index (EI) was determined. The EI was calculated according to the following formula: EI = diameter of hydrolytic zone / colony diameter (mm), as described by Saroj and Narasimhulu [43]. For EI > 1, the activity was considered good; for EI = 1–2, the activity was very good; and for EI > 2, the activity was considered high. The amylase, protease, xylanase, and mannanase production of sugar beet isolates was performed as previously described [44]. Lipolytic activity was tested on plates containing TSA and 1% glycerol tributyrate, while production was confirmed by appearance of the illuminated zones around bacterial growth [45]. Furthermore, sugar beet isolates were evaluated for their ability to produce cellulases [46], gelatinases [47], and pectinases [48].

### Stress Tolerance of Endophytic and Rhizospheric Sugar Beet Bacteria *In Vitro*

Salinity stress tolerance was evaluated by the modification of the method described by Gupta et al. [39]. NA plates, previously enriched with 1%, 3%, 5%, 8%, and 10% NaCl, were spot inoculated with 5 μL of the overnight bacterial culture and incubated for 24 h at 30 °C, after which the growth of isolates was evaluated. The results are presented using positive and negative symbols relative to the growth observed at 1% NaCl in the substrate. For drought tolerance, a method adapted from Ali et al [49] was used. Overnight cultures were grown in diluted TSB medium (10 g/L), and after measuring the optical density (OD600), all isolates were diluted to 0.4. The tolerance of strains to desiccation was studied in TSB medium (10 g/L) amended with 5, 10, 20, and 30% PEG in flasks containing 1% bacterial cultures (OD_600_ = 0.4). The flasks were incubated overnight at 30 °C (150 rpm). Evaluation of the percent growth of isolates in the various concentrations of PEG was performed in comparison to a control, with the optical density of the control (OD_600_) set at a value of 100%. These percentage results were then labelled with symbols corresponding to the different concentration intervals: + for the 0–30% range, ++ for the 30–60% range, and +++ for the 60–100% range.

### Antifungal Potential of Endophytic and Rhizospheric Sugar Beet Bacteria Against Phytopathogenic Fungi *In Vitro*

The antifungal potential against the 22 phytopathogenic fungi was evaluated using the initial screening and co-culture plate test [50]. The fungal pathogens tested were *Fusarium* sp. TS1, *Fusarium oxysporum* TS2, *Fusarium equiseti* TS3, *Fusarium subglutinans* TS5, *Fusarium nigamai* TS6, *Fusarium solani* TS8, and *Cercospora beticola* TS4 from sugar beet as well as *Fusarium semitectum* TS7, *F. graminearum* GD1, *F. graminearum* S3-7, *F. graminearum* CIK, *F. oxysporum* S4-2, *F. verticillioides* K67 5.1, *F. venatum* IB1I-12, *F. ipomoeae* IB6I-10, *F. foetens* IP27, *F. falciforme* IP31, *F. coffeatum* IP32, and *F. denticulatum* IP39 from different origins. The origin of all phytopathogenic fungi is listed in Table S1. The existing collection is part of the Department of Algology and Mycology, Institute of Botany and Botanical Garden “Jevremovac,” University of Belgrade — Faculty of Biology. Effects on mycelial growth were evaluated by calculating the percent of growth inhibition, *PGI* % = 100(*KR* − *R*1)/*KR*, where *KR* represents the distance (measured in mm) from the point of inoculation to the colony margin on the control dishes, and R1 is the distance of fungal growth from the point of inoculation to the colony margin on the treated dishes in the direction of the antagonist [50]. The experiments were repeated twice independently, with three replications for each fungus. Following the Kolmogorov–Smirnov test for normality, standard variance analysis was used to analyze the obtained PGI% results (one-way ANOVA test). *In vitro* mycelial growth inhibition percentages were averaged out using the Tukey’s HSD test. Statistical significance applied in all tests was *p* < 0.05. The statistical analysis was performed according to the general procedures of IBM SPSS Statistics v.23 (SPSS, Inc., Armonk, NY, USA).

## Results

### The Analysis of Alpha and Beta Diversities of the Seed Endophytic Bacteria

**Fig. 2:**
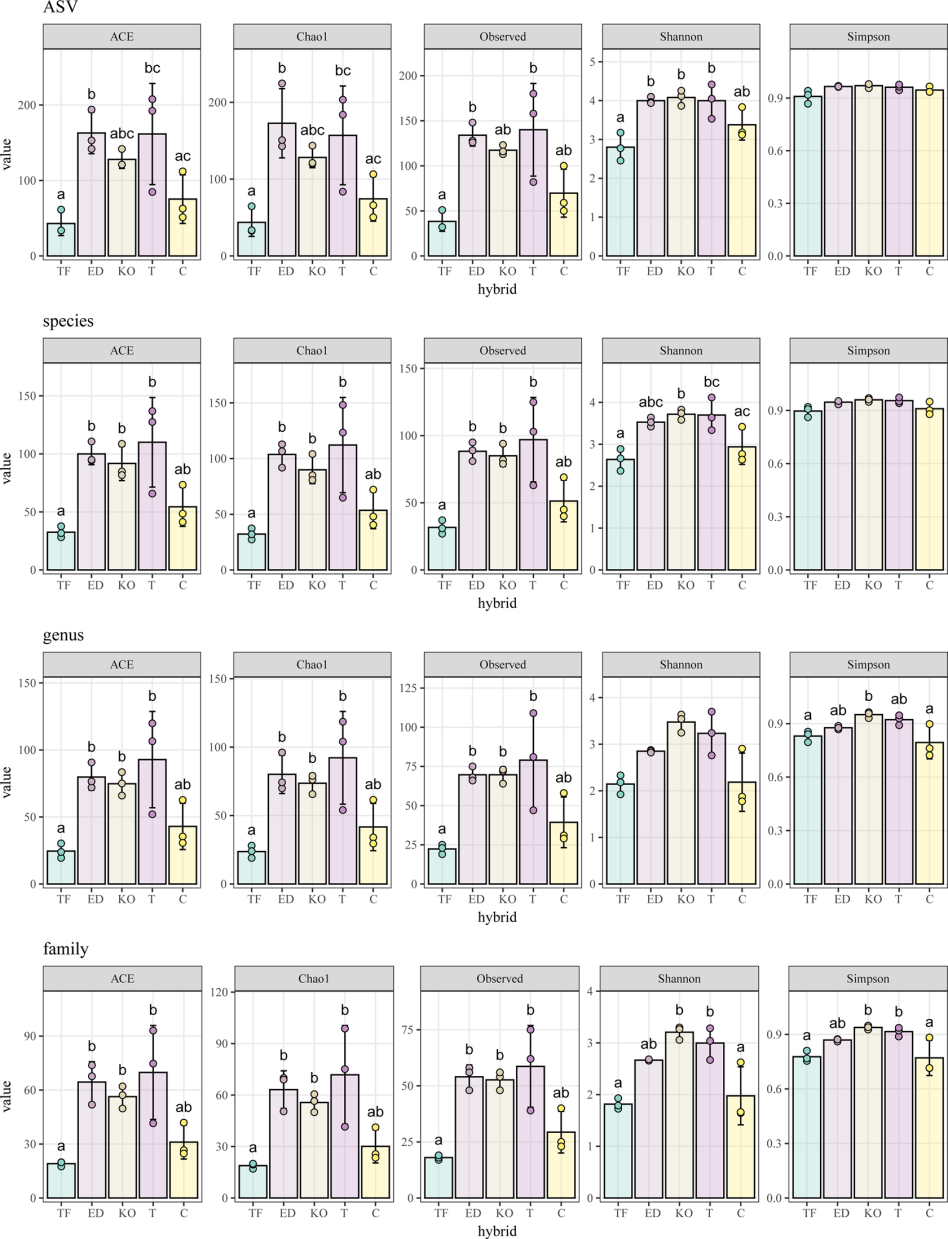
The alpha diversity in the five hybrids Tajfun (TF), Eduarda (ED), Koala (KO), Tibor (T), and *Cercospora*-resistant (C) was compared at all tax-level using Kruskal–Wallis one-way analysis of variance, while a Dunn post hoc test was run for pairwise group comparison. The values marked with the same letter within the diagram columns do not indicate statistically significant differences

**Fig. 3:**
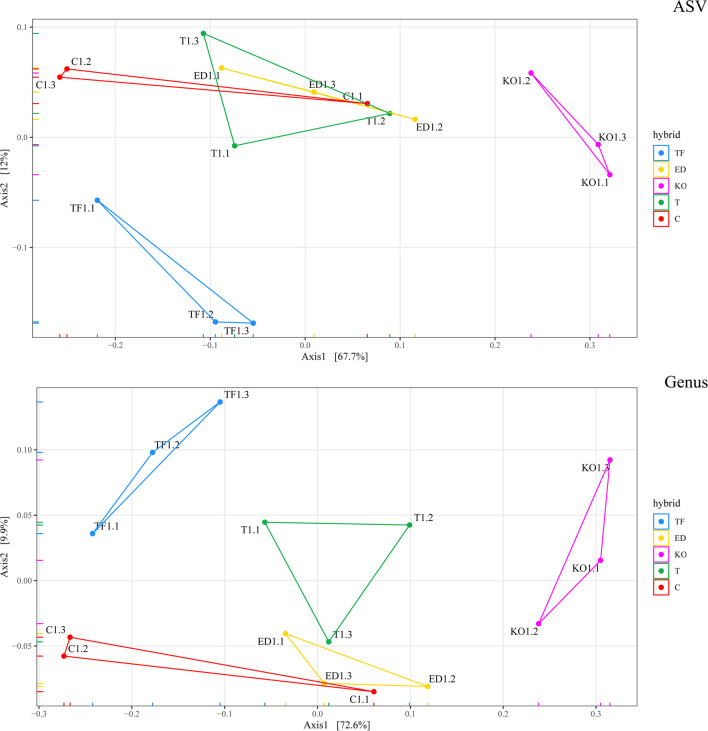
Beta diversity among five sugar beet hybrids Tajfun (TF), Eduarda (ED), Koala (KO), Tibor (T), and *Cercospora*-resistant (C) presented by DPCoA at the ASV and genus levels

The phylogenetic composition of bacterial communities associated with seeds of five sugar beet cultivars was analyzed by amplification and sequencing of the V3–V4 region of the 16S rRNA gene. The average number of amplicon sequence variants (ASVs) after removal of chimeras per hybrid was 75838.33 (C), 71714.33 (ED), 77315.67 (KO), 73495.00 (T), and 75020.67 (TF) (Table S6). Alpha diversity indexes were calculated for the number of observed features (OBS), Chao1, ACE, and Shannon and Simpson at family, genus, species, and ASV levels (Fig. 2). Higher alpha diversity was evident in ED, KO, and T hybrid across all taxonomic levels, in contrast to C and TF hybrid, with the observed richness showing correlation with estimated richness, except at the ASV level. ED hybrid displayed the highest richness based on Chao1 and ACE indices at the ASV level, while both ED and T hybrids exhibited the highest observed richness according to OBS. Conversely, TF hybrid consistently exhibited the lowest richness at all taxonomic levels based on both estimated and observed richness estimators. Shannon and Simpson indices indicated no statistically significant changes in alpha diversity at the family level between TF and C hybrid and between KO and T hybrid. However, at the species level, statistically significant differences were observed between all hybrids, with Shannon indices being the discriminant factor. On the other hand, at the ASV level, no statistically significant differences were found between ED, KO, and T hybrids, whereas C and TF hybrids showed significant differences from all other hybrids. The PCoA plot demonstrated that KO hybrid at the ASV level exhibited separation from the other hybrids along axis 1, explaining 67.7% of the variability, while ED, C, and T hybrids were grouped together (Fig. 3). TF hybrid showed distinct separation from the others along axis 2, accounting for 12% of the variance. Better separation of hybrids was observed at the genus level. The first axis (explaining 72.6% of variability) indicated that C and TF were distinct from the remaining hybrids based on genus composition, with KO hybrid being the most distant from all hybrids. Additionally, along the second axis (explaining 9.9% of variability), a clear separation of C hybrid from TF hybrid and the other hybrids, as well as its association with ED hybrid, was observed. At the genus and ASV levels, KO and TF appeared to be more distant from the remaining hybrids. PERMDISP2 results revealed a non-significant difference in hybrids dispersions at all taxonomic levels. However, the PERMANOVA test showed statistically significant variability between hybrids (*p* = 0.001) at all taxonomic levels.

### The Composition of the Seed Endophytic Bacteria Determined Using the NGS Approach

**Fig. 4:**
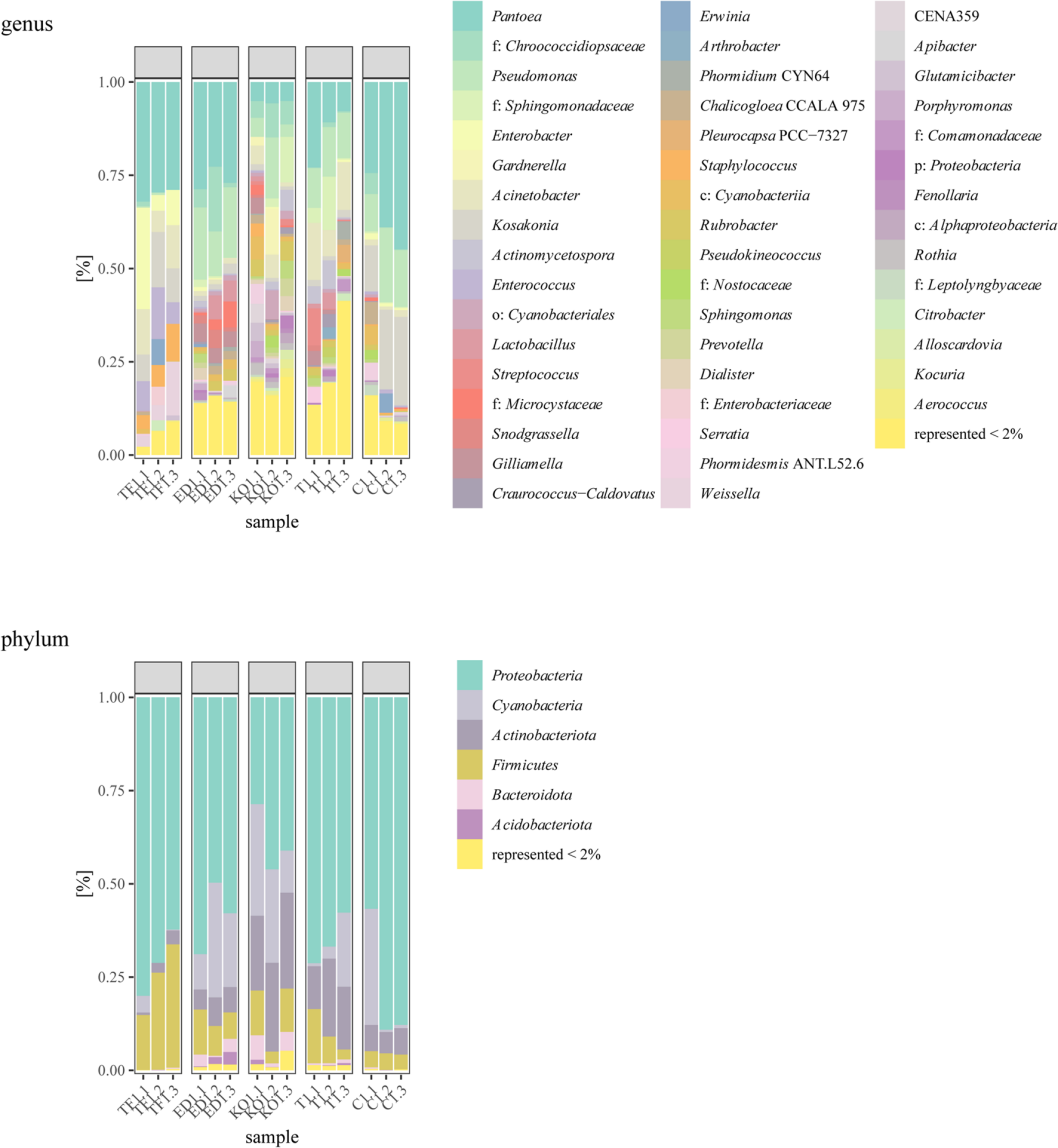
Relative abundance of bacterial phylum and genera associated with seeds from five sugar beet hybrids Tajfun (TF), Eduarda (ED), Koala (KO), Tibor (T), and *Cercospora*-resistant (C)

Of the 16 phyla detected, the phylum *Proteobacteria* was the most widespread in all five hybrids with relative abundance (RA) ranging from 38.63% in the KO hybrid up to 77.90% in the C hybrid (Fig. 4). The following major phyla in all hybrids were *Cyanobacteria* and *Actinobacteriota*, except in the TF hybrid. A highest representation of the phylum *Firmicutes* was detected in the TF hybrid with a RA of 24.65%, while *Bacteroidota* was the most abundant in the KO (4.20%). The most prevalent genus was *Pantoea* detected in the C, ED, and TF hybrids. *Pseudomonas* was the second most abundant genus in all hybrids, except for TF. In general, TF hybrid was characterized by *Pantoea* (30.23%), *Enterobacter* (13.63%), *Kosakonia* (10.35%), *Acinetobacter* (9.82%), *Enterococcus* (9.22%), *Weissella* (7.14%), *Staphylococcus* (6.47%), and *Erwinia* (2.31%). Genera *Gardnerella* (4.88%), *Rubrobacter* (3.71%), *Glutamicibacter* (2.28%), *Sphingomonas* (2.24%), *Rothia* (2.18%), and *Prevotella* (2.02%) were most abundant in KO hybrid. Most dominant genera in the T hybrid were *Pantoea* (13.87%), *Pseudomonas* (12.09%), and *Acinetobacter* (11.74%), followed by *Actinomycetospora* and *Streptococcus* (4.25% and 3.38%, respectively). The C hybrid had the lowest genera diversity, and it was dominated by *Pantoea* (36.09%), *Kosakonia* (19.15%), and *Pseudomonas* (15.10%). Most detected genera in ED were *Pantoea* (26.17%), *Pseudomonas* (16.76%), *Gilliamella* (4.37%), and *Lactobacillus* (4.11%) and two unidentified belonging to the families *Chroococcidiopsaceae* and *Microcystaceae*, with 7.81% and 3.48%, respectively. The genus *Songrasella* (2.72%) was most abundant in ED.

**Fig. 5:**
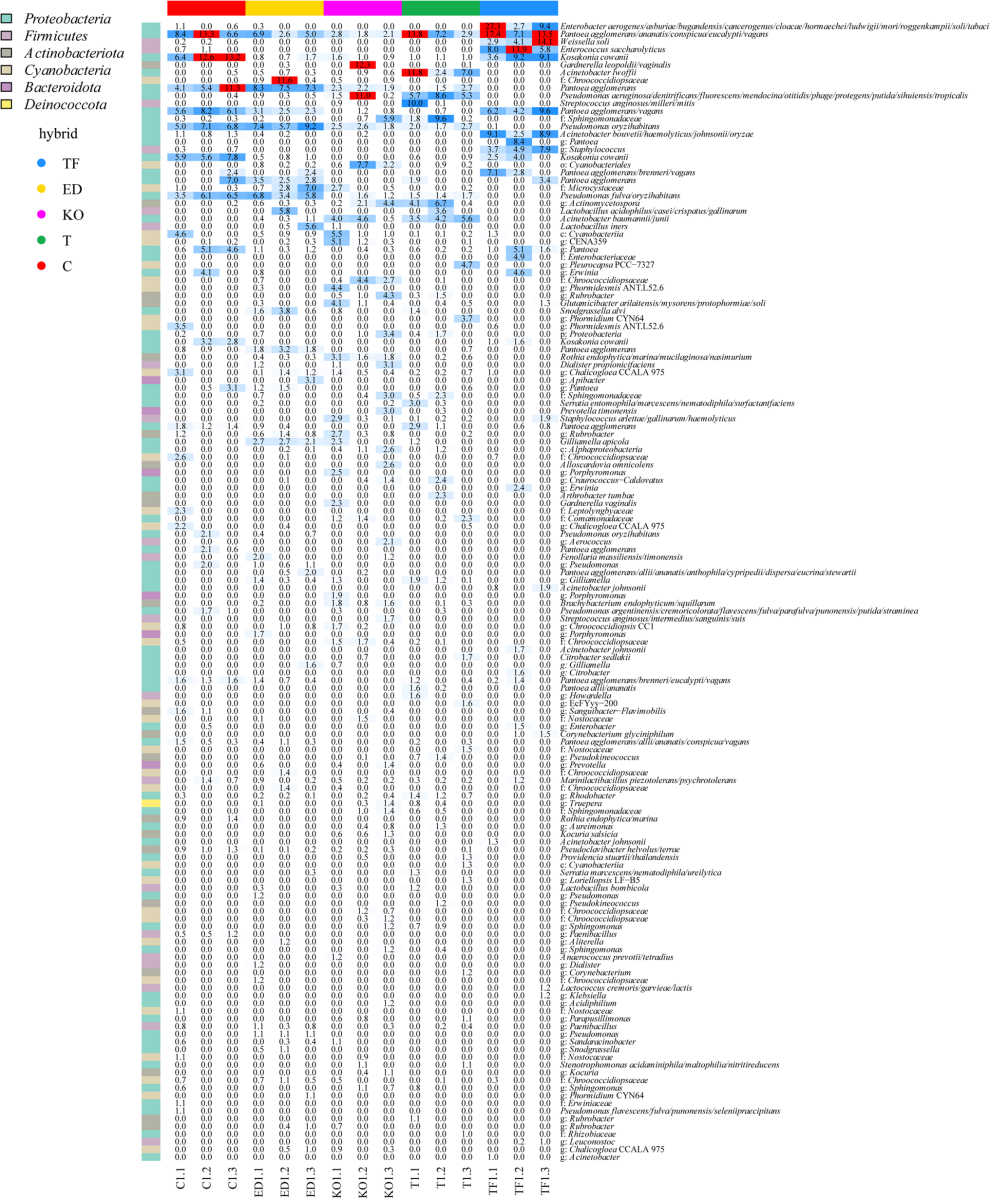
Heatmap of relative abundance of bacterial ASVs associated with the with seeds from five sugar beet hybrids Tajfun (TF), Eduarda (ED), Koala (C), Tibor (T), and *Cercospora*-resistant (C)

The highest average reads of *Pantoea agglomerans/ananatis/conspicua/eucalypti/vagans* at ASV level were recorded for TF, C, and T (Fig. 5). *Kosakonia cowanii* with the highest percentage of reads was detected in C and TF, while its abundance was significantly reduced in ED, KO, and T hybrids. *Acinetobacter lwoffii* was detected in T hybrid. Furthermore, ASV related to *Acinetobacter baumannii/junii* had a similar abundance in T and KO, while *Acinetobacter bouvetii/haemolyticus/johnsonii/oryzae* was characteristic for the TF. *Weissella* was highly detected in the TF hybrid with 7% of reads assigned as *Weissella soli*. *Enterococcus saccharolyticus* was highly presented in the TF hybrid. A higher percentage of ASV reads for *Pseudomonas oryzihabitans* and *Pseudomonas fulva/oryzihabitans* was detected in C and ED hybrid.

### The Differential Abundance Analysis of the Seed Endophytic Bacteria Between Hybrids

**Fig. 6:**
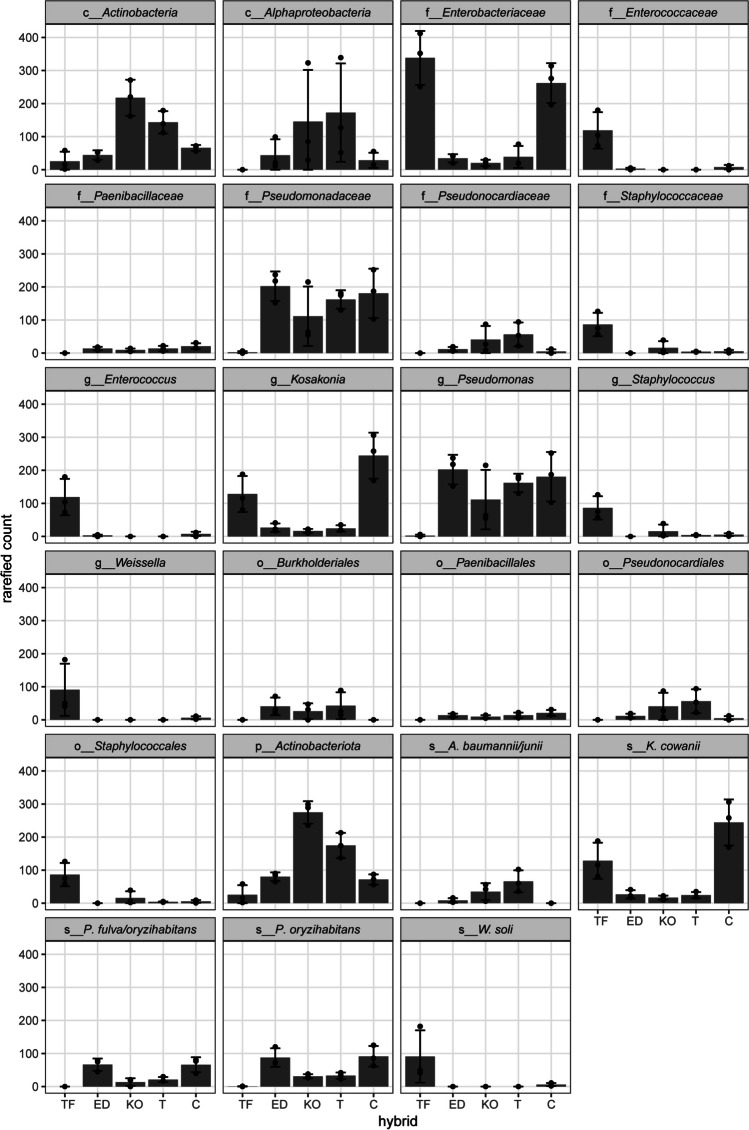
Differential abundance analysis of significantly more abundant taxa among in the seeds of five sugar beet hybrids Tajfun (TF), Eduarda (ED), Koala (KO), Tibor (T), and *Cercospora*-resistant (C). Bonferroni method was used for *p*-adjustment, and values of *p* < 0.01 were considered to be statistically significant

The differential abundance estimation identified 23 taxa that were significantly more abundant in some hybrids compared to others (Fig. 6). Specifically, *Actinobacteria* and *Alphaproteobacteria* were found to be significantly more abundant in KO and T hybrids. On the other hand, families such as *Pseudomonadaceae*, *Paenibacillaceae*, *Pseudonocardiaceae*, *Enterococcaceae*, and *Staphylococcaceae* exhibited statistically different representation in the hybrids. Notably, TF and C hybrids exhibited a significantly higher presence of the *Enterobacteriaceae* family compared to the other hybrids. Family *Pseudomonadaceae* was detected in all samples but displayed a statistically higher abundance in ED, KO, T, and E hybrids compared to TF hybrid. Similarly, *Paenibacillaceae* showed greater abundance in T and C hybrids, while *Pseudonocardiaceae* was more prevalent in KO and T hybrids. *Enterococcaceae* and *Staphylococcaceae* were found to be the most abundant families in TF hybrid, in comparison to the other hybrids. The separation of hybrids was influenced by the orders *Burkholderiales*, *Paenibacillales*, *Pseudonocardiales*, and *Staphylococcales*. *Burkholderiales* showed statistical enrichment in ED, KO, and T hybrids, while *Paenibacillales* was the characteristic of all hybrids when compared to TF hybrid. *Pseudonocardiales* was more prevalent in KO and T hybrid, whereas *Staphylococcales* dominated in TF hybrid. Additionally, specific genera like *Weissella*, *Staphylococcus*, and *Enterococcus* were significantly present only in TF hybrid. Additionally, *W. soli* exhibited the same pattern with highest abundance in TF hybrid. The genus *Kosakonia* with species *K. cowanii* displayed statistical significance in abundance in TF and C hybrids compared to other hybrids, with its highest prevalence observed in C hybrid. Furthermore, the genus *Pseudomonas* showed higher prevalence in ED and KO compared to other hybrids, and this pattern was consistent for *P. oryzihabitans*. In summary, the differential abundance analysis revealed distinct taxonomic differences among the hybrids, indicating significant variations in the representation of specific taxa and genera, thus providing valuable insights into the microbial composition and diversity in the studied populations.

### Diversity of Cultured Endophytic (Seed and Roots) and Rhizospheric Bacteria of Sugar Beet Hybrids

Each sugar beet hybrid’s rhizosphere contained species specific only to that particular hybrid, with one common species shared among all hybrids (Fig. 1). The isolates obtained from the roots of the hybrids did not exhibit any shared species. Notably, the root of the T hybrid displayed the highest species diversity, with 13 species unique to this hybrid. Comparing different hybrids, the roots of ED, KO, and C hybrids shared one or two species solely with the T hybrid. The ED hybrid showed the highest seed diversity, followed by C and KO hybrids with 12, 11, and 10 unique species, respectively. A total of 156 isolates were obtained from all hybrids, comprising 22, 31, and 63 unique species from the rhizosphere, roots, and seeds, respectively (Table S2).

The rhizosphere of sugar beet hybrids showed *Lysinibacillus macroides* to be a fundamental species, serving as the core member of this ecological niche. Among the *Lysinibacillus* genus, *L. macroides*, *L. pakistanensis*, and *L. fusiformis* were specifically isolated from the rhizosphere of the TF hybrid (Table 1; Table S3). The roots were particularly abundant in the genera *Paenibacillus*, *Curtobacterium*, *Mycetocola*, *Knoellia*, *Neorhizobium*, *Microbacterium*, *Rhodococcus*, *Massilia*, and *Rathayibacter*, with a total of 93 strains. The seeds displayed the highest diversity, especially in the case of ED (Table S3), KO (Table S3), and C (Table S3) hybrids. *Bacillus subtilis* was a unique species found in the seeds of all hybrids (Table 1). Additionally, prominent seed endophytes included species from the genera *Bacillus*, *Lysinibacillus*, *Kocuria*, *Sanguibacter*, *Pantoea*, *Glutamicibacter*, *Pseudomonas*, *Erwinia*, *Providencia*, and *Pseudoclavibacter*. Moreover, *Curtobacterium pusillum* was a unique species for C, ED, and KO seeds, while *Bacillus sonorensis* was isolated exclusively from the seeds of the T hybrid (Table 1; Table S3). Analyzing the diversity of culturable bacteria in the rhizosphere, roots, and seeds of each hybrid individually revealed minimal species overlap.

**Table 1:** The overlap of culturable bacteria of five sugar beet hybrids from the rhizosphere, roots, and seeds

Common species in hybrids	No. of unique species	Species
Rhizosphere		
*Cercospora-*resistant and Eduarda and Koala and Tajfun and Tibor	1	*Lysinibacillus macroides*
Eduarda and Tajfun	1	*Lysinibacillus fusiformis*
Tajfun and Tibor	1	*Lysinibacillus pakistanensis*
Koala and Tibor	1	*Bacillus wiedmannii/proteolyticus/fungorum*
*Cercospora-*resistant and Koala	1	*Bacillus pacificus/paranthracis*
Eduarda	2	*Bacillus badius*; *Cytobacillus ciccensis*
Tibor	2	*Bacillus altitudinis/aerophilus/stratosphericus*; *Priestia aryabhattai*
Tajfun	1	*Bacillus mobilis*
Koala	2	*Brevibacillus reuszeri*; *Rossellomorea marisflavi*
*Cercospora-*resistant	2	*Bacillus toyonensis/thuringiensis*; *Brevibacillus borstelensis*
Root endophytes		
Eduarda and Tibor	1	*Bacillus velezensis*
Koala and Tibor	1	*Micrococcus aloeverae*
*Cercospora-*resistant and Tibor	2	*Massilia timonae*; *Micrococcus luteus*
Eduarda	5	*Curtobacterium pusillum*; *Knoellia locipacati*; *Microbacterium thalassium*; *Mycetocola manganoxydans*; *Paenibacillus taichungensis*
Tibor	13	*Arthrobacter oryzae*; *Bacillus halotolerans*; *Kocuria rosea*; *Metabacillus indicus*; *Microbacterium testaceum*; *Neobacillus niacini*; *Neorhizobium huautlense*; *Paenibacillus azotifigens*; *Paenibacillus catalpae/lupini*; *Paenibacillus lautus*; *Rathayibacter tritici*; *Rhodococcus cerastii*; *Rhodococcus corynebacterioides*
Tajfun	2	*Bacillus subtilis*; *Micrococcus luteus/aloeverae*
Koala	2	*Micrococcus terreus*; *Staphylococcus epidermidis*
*Cercospora-*resistant	1	*Bacillus pseudomycoides*
Seed endophytes		
*Cercospora-*resistant and Eduarda and Koala and Tajfun and Tibor	1	*Bacillus subtilis*
*Cercospora-*resistant and Eduarda and Koala	2	*Bacillus zhangzhouensis*; *Curtobacterium pusillum*
Eduarda and Tibor	1	*Acinetobacter lactucae*
Eduarda and Koala	3	*Kosakonia cowanii*; *Micrococcus aloeverae*; *Pseudomonas oryzihabitans*
*Cercospora-*resistant and Eduarda	1	*Bacillus mobilis*
*Cercospora-*resistant and Koala	1	*Pantoea agglomerans/Curtobacterium plantarum*
Eduarda	12	*Alkalihalobacillus rhizosphaerae*; *Erwinia persicina*; *Erwinia tasmaniensis*; *Frigoribacterium faeni*; *Glutamicibacter mishrai*; *Klebsiella michiganensis*; *Microbacterium testaceum*; *Micrococcus luteus*; *Providencia sneebia*; *Providencia vermicola*; *Sanguibacter keddieii*; *Staphylococcus succinus* subsp. *succinus*
Tibor	1	*Bacillus sonorensis*
Tajfun	6	*Bacillus licheniformis*; *Bacillus oleivorans*; *Brevundimonas nasdae/vesicularis*; *Microbacterium paludicola*; *Paenibacillus lautus*; *Weizmannia ginsengihumi*
Koala	10	*Alkalihalobacillus gibsonii*; *Bacillus haynesii*; *Enterococcus gallinarum*; *Microbacterium arborescens/imperiale*; *Microbacterium saccharophilum*; *Mixta theicola*; *Okibacterium fritillariae*; *Pantoea agglomerans*; *Pantoea allii*; *Sanguibacter inulinus*
*Cercospora*-resistant	11	*Bacillus altitudinis/aerophilus/stratosphericus*; *Bacillus amyloliquefaciens*; *Bacillus halotolerans*; *Bacillus mojavensis*; *Bacillus pumilus/zhangzhouensis/safensis*; *Bacillus wiedmannii/proteolyticus/fungorum*; *Corynebacterium doosanense*; *Lysinibacillus macroides*; *Lysinibacillus pakistanensis*; *Pseudoclavibacter helvolus*; *Paenibacillus polymyxa*

### Evaluation of the Plant Growth–Promoting Properties *In Vitro* of Cultured Endophytic (Seed and Roots) and Rhizospheric Bacteria of Sugar Beet Hybrids

Examination of extracellular enzyme production and PGP characteristics (nitrogen fixation, phosphate solubilization, EPS production, swarming and swimming ability) was initially performed on 156 isolates (Table S4). The vast majority of isolates were able to grow on nitrogen-free media (86%), while 38% were phosphate-solubilizing bacteria. Swimming motility was recorded in a larger number of isolates (69%) in relation to the form of swarming (44%). Production of EPS was exhibited by 46 isolates (29%). Gelatinases were the most commonly secreted enzymes (44%), followed by amylases (30%), mannanases (17%), proteinases (17%), cellulases (16%), and pectinases (14%). Xylanase production was achieved by a significantly limited proportion of isolates, representing only 6% of the total. Isolates with a good and wider spectrum of PGP ability and secreted enzymes were selected for further testing on additional PGP characteristics (production of IAA, HCN, ACC deaminase, lipase and siderophores, drought and salinity tolerance).

Preliminary screening of sugar beet isolates for plant growth ability resulted in the selection of 32 bacterial strains with good PGP characteristics, while potential pathogens were classified as risk group 2 and excluded from the selection (Table 2).

**Table 2:** PGP abilities and enzymatic activity for the best selected strains

Species	Strain	Origin	Growth +N_2_	Growth -N_2_	Sol P	IAA	Sider	EPS	Swar	Swim	HCN	ACC	Amy	Prot	Xyl	Mann	Cell	Gel	Pect	Lip
													EI	EI	EI	EI	EI	EI	EI	EI
*Bacillus halotolerans*	C3-16/2.1	Seed	+ +	+ +	+	─	+ +	─	+ + +	+ + +	+	─	1.2	1.8	─	1.5	1.8	1.3	2.0	1.1
*Bacillus amyloliquefaciens*	C3-19	Seed	+ + +	+	+	─	+ +	─	+ + +	+ + +	─	─	─	1.2	─	1.7	1.6	1.6	1.6	─
*Paenibacillus polymyxa*	C3-36	Seed	+ +	+ +	─	+	─	+	─	+ +	─	─	1.6	─	1.6	1.2	2.0	1.4	1.3	─
*Bacillus zhangzhouensis*	C3-50	Seed	+	+	+	─	+	+ +	+	+ + +	+	─	─	1.2	─	─	─	1.7	─	1.6
*Lysinibacillus macroides*	C3-53	Seed	+ +	+	+	+	─	─	+ + +	+ + +	─	─	─	─	─	─	─	─	─	1.3
*Bacillus subtilis*	C3-59	Seed	+ +	+ +	+	─	+ + +	─	+ +	+ + +	─	─	1.4	1.1	─	─	─	1.4	─	─
*Bacillus subtilis*	C3-62	Seed	+ +	+	─	─	+ + +	+	─	+ + +	─	─	─	─	─	1.4	─	1.5	1.8	─
*Bacillus subtilis*	C3-72	Seed	+ +	+	+	─	+	+	+ + +	+ + +	+ +	─	1.5	1.6	─	─	1.7	1.5	─	1.2
*Paenibacillus taichungensis*	ED2-1	Root	+ +	+	─	+	+	─	─	+ + +	─	─	2.5	─	1.1	─	1.3	1.1	1.3	1.2
*Bacillus velezensis*	ED2-2	Root	+ +	+	─	─	+ +	─	+ + +	+ + +	─	─	1.3	1.3	─	1.7	1.2	1.9	1.5	─
*Curtobacterium pusillum*	ED2-6	Root	+	+	+	+	+ +	─	+	+ +	+ +	+	─	─	─	─	─	1.8	─	1.2
*Providencia vermicola*	ED3-10	Seed	+ +	+	+	+ + +	+	─	+	+ + +	─	─	─	─	─	─	─	─	─	─
*Glutamicibacter mishrai*	ED3-75	Seed	+ +	+	─	+	+	─	─	─	+ + +	─	1.8	2.0	─	1.2	─	1.9	─	1.3
*Erwinia tasmaniensis*	ED3-79	Seed	+	+ +	+	+	+ +	+ + + ^d^	+ + +	+ + +	+ + +	─	─	─	─	─	─	─	─	─
*Bacillus subtilis*	ED3-89	Seed	+	+ +	+	+	+	─	+ + +	+ + +	─	─	─	─	─	1.1	1.6	1.5	1.2	1.1
*Bacillus pacificus/paranthracis*	KO1-1	Rhiz.	+	+	+	─	+	─	+	+ + +	─	─	1.6	─	─	─	1.3	2.1	─	─
*Bacillus subtilis*	KO3-11/1	Seed	+ +	+ +	+	+	─	+	+ + +	+	─	─	1.2	─	─	─	1.2	1.3	2.4	1.0
*Bacillus subtilis*	KO3-18	Seed	+	+ +	+	─	+ + +	+	+ + +	+ + +	+	+	1.1	─	─	─	1.8	1.4	2.4	1.3
*Pseudomonas oryzihabitans*	KO3-19	Seed	+ +	+ +	+ +	─	─	+	+ + +	+ + +	+ +	─	─	─	─	─	─	─	─	1.8
*Bacillus subtilis*	KO3-26	Seed	+ +	+	+	─	+	─	+ + +	+ + +	─	─	1.6	─	─	─	1.1	1.7	2.1	─
*Mixta theicola*	KO3-44	Seed	+ +	+ + +	+	+ + +	+	+ + + ^d^	+	+ + +	─	─	─	─	─	─	─	─	─	─
*Priestia aryabhattai*	T1-2	Rhiz.	+ +	+	+	+	+	+	+	─	─	+	─	─	─	─	─	1.9	─	1.1
*Bacillus halotolerans*	T2-1	Root	+ +	+	+	─	+ +	─	+ + +	+ + +	─	─	1.7	1.2	─	1.8	2.2	1.8	─	─
*Bacillus velezensis*	T2-23	Root	+ +	+	─	─	+ + +	─	+ + +	+ + +	─	─	1.4	1.2	─	1.8	1.5	1.7	1.7	─
*Arthrobacter oryzae*	T2-25	Root	+	+ +	─	+	─	─	─	─	─	─	1.3	─	1.5	1.4	─	1.7	─	─
*Micrococcus aloeverae*	T2-26	Root	+ +	+	─	+ +	─	─	+	+	─	─	─	─	─	─	─	1.7	─	─
*Bacillus subtilis*	T3-4	Seed	+	+ +	+	+	+ +	─	+ + +	+ + +	+ +	─	1.3	1.2	─	1.8	1.8	1.5	2.0	1.3
*Bacillus sonorensis*	T3-5	Seed	+ +	+	+	+	+	─	+ + +	+ + +	─	─	1.3	─	1.3	1.4	2.0	1.2	─	─
*Bacillus subtilis*	TF2-1	Root	+ +	+ +	+	─	+	+	+ + +	+ + +	─	─	1.1	1.1	─	─	─	1.6	─	─
*Bacillus subtilis*	TF3-6	Seed	+	+ +	+	─	+ +	+	+ + +	+ + +	─	─	1.7	1.1	2.3	─	─	1.5	2.3	1.1
*Bacillus subtilis*	TF3-7/1	Seed	+ +	+	+	─	+	+	+ + +	+ + +	─	─	1.1	─	2.0	1.4	─	1.8	1.9	─
*Bacillus subtilis*	TF3-32	Seed	+ + +	+ +	─	─	+	─	+ + +	+ + +	─	─	1.4	1.1	─	─	2.2	1.9	─	─

Eduarda (ED), Koala (KO), Tibor (T), Tajfun (TF), and *Cercospora*-resistant (C) — numbers beside hybrid abbreviations indicate the origin of the isolate, i.e., rhizosphere (1), root (2), and seed (3)

─ no activity; + weak activity; ++ good activity; +++ very good activity; +++^d^ excellent activity. *EI* enzymatic index (EI >1 good activity; EI = 1–2 very good activity; EI > 2 excellent activity), *Sol P* phosphate solubilization, *IAA* indole-3-acetic acid (IAA) production, *Sider* siderophore production, *EPS* exopolysaccharides production, *Swarm* swarming motility, *Swim* swimming motility, *HCN* HCN production, *ACC* 1-aminocyclopropane-1-carboxylic acid (ACC) deaminase activity, *Amy* amylases, *Prot* proteases, *Xyl* xylanases, *Mann* mannanases, *Cell* cellulases, *Gel* gelatinases, *Pec* pectinases, *Lip* lipases

All 32 isolates had the ability to grow on nitrogen-free media, whereas some isolates exhibited more intensive growth, especially *Mixta theicola* KO3-44, indicating better nitrogen fixation abilities. The most remarkable solubilization capacity observed in *P. oryzihabitans* KO3-19. Isolate *B. subtilis* KO3-18 had the highest PGP potential under *in vitro* conditions. Excellent producers of IAA were *Providencia vermicola* ED3-10 and *M. theicola* KO3-44. The best siderophore production was recorded for *B. subtilis* KO3-18, *B. subtilis* C3-59, *B. subtilis* C3-62, and *Bacillus velezensis* T2-23. ACC deaminase production was observed only for *B. subtilis* KO3-18, *C. pusillum* ED2-6, and *Priestia aryabhattai* T1-2. Significant production of EPS was recorded for *M. theicola* KO3-44 and *Erwinia tasmaniensis* ED3-79, while *E. tasmaniensis* ED3-79 and *Glutamicibacter mishrai* ED3-75 showed the greatest potential in HCN production.

### Evaluation of the Exoenzymatic Activity *In Vitro* of Cultured Endophytic (Seed and Roots) and Rhizospheric Bacteria of Sugar Beet Hybrids

The most frequently secreted enzyme was gelatinase detected in 27 isolates with recorded EI values from 1.1 to 2.1, whereas the highest value was exhibited by *Bacillus pacificus/paranthracis* KO1-1. Twenty isolates showed amylolytic activity (1.1–2.5) with the highest activity observed in *Paenibacillus taichungensis* ED2-1. For cellulase production with EI range from EI 1.1 to 2.2, peak cellulase activity was exhibited by *B. subtilis* TF3-32. Among 14 isolates that were able to degrade the pectin (1.2–2.4), *B. subtilis* KO3-18 had the highest pectinolytic activity. Lipolytic activity was observed in the same number of isolates (1.1–1.8), and *P. oryzihabitans* KO3-19 stood out with the highest EI index value. Extracellular production of proteinases (1.1–2.0) and mannanases (1.1–1.8) were recorded for 13 isolates; and. *G. mishrai* ED3-75 and *B. velezensis* T2-23 exhibited the highest activity. The xylanolytic activity was recorded only for six isolates with EI values in the range 1.7–2.3, with the highest activity observed in *B. subtilis* TF3-6. Isolates *B. velezensis* T2-23, *B. subtilis* T3-4, *B. velezensis* ED2-2, and *Bacillus halotolerans* C3-16/2.1 were positive for all tested enzymatic activity, except for xylanase production. For *Paenibacillus polymyxa* C3-36, only proteolytic activity was missing. In contrast, isolates identified as *L. macroides*, *M. theicola*, *P. vermicola*, *P. oryzihabitans*, and *E. tasmaniensis* did not exhibit any exoenzymatic activity.

### Assessment of Salt and Drought Tolerance *In Vitro* of Cultured Endophytic (Seed and Roots) and Rhizospheric Bacteria of Sugar Beet Hybrids

The majority of isolates exhibited successful growth up to an 8% NaCl concentration (Table S5). Out of 32 tested, 24 isolates could tolerate 8% NaCl, while half of them grew at 10% NaCl. *B. halotolerans* C3-16/2.1 exhibited the greatest tolerance to salinity, even at 10% NaCl. Almost all 32 isolates successfully tolerated drought in 5% and 10% PEG concentrations (Table S5). Eighteen isolates showed a tolerance range from moderate to complete resistance when exposed to a medium containing 20% PEG. Of the 32 isolates, two grew successfully at the maximum concentration of PEG with no visible reduction in growth compared to the control (*B. subtilis* C3-62 and *B. subtilis* TF2-1), while the growth of *B. halotolerans* C3-16/2.1, *Bacillus amyloliquefaciens* C3-19, *Bacillus zhangzhouensis* C3-50, *B. velezensis* ED2-2, *B. velezensis* T2-23, *M. aloeverae* T2-26, and *B. sonorensis* T3-5 was reduced to 50% of the control.

### Evaluation of Antifungal Activity *In Vitro* of Cultured Endophytic (Seed and Roots) and Rhizospheric Bacteria of Sugar Beet Hybrids

According to the results obtained for PGP potential and exoenzymatic activity, 60 isolates were selected for initial screening against *Fusarium* sp. TS1, *Fusarium equiseti* TS2, *Fusarium oxysporum* TS3, and *Cercospora beticola* TS4 obtained from infected sugar beets. For further screening of antagonistic activity using the dual cultivation method, 32 isolates were selected from an original 60 that showed good inhibitory activity in the initial screening. Out of 32 isolates, 19 isolates showed moderate to exceptional antagonistic activity against all tested fungi (Table 3).

**Table 3:** Antifungal activity (mean ± SE) of selected isolates on fungi from infected sugar beet *in vitro* using a method of dual cultivation

Species	Strain	*Fusarium* sp. TS1	*Fusarium equiseti* TS2	*Fusarium oxysporum* TS3	*Cercospora beticola* TS4
*Cercospora-*resistant					
*Bacillus halotolerans*	C3-16/2.1	35.6 ± 0.79^cdefg^	51.0 ± 0.58^bc^	42.0 ± 2.14^defghi^	52.3 ± 1.31^bcd^
*Bacillus amyloliquefaciens*	C3-19	42.5 ± 1.58^abcde^	53.0 ± 2.89^b^	46.9 ± 0.71^bcde^	**68.9 ± 0.95**^**a**^
*Paenibacillus polymyxa*	C3-36	49.3 ± 2.37^ab^	**67.0 ± 1.73**^**a**^	**55.6 ± 1.42**^**a**^	52.3 ± 1.31^bcd^
*Bacillus zhangzhouensis*	C3-50	11.0 ± 5.54^jkl^	34.0 ± 2.31^ef^	11.1 ± 2.85^l^	29.5 ± 1.31^gh^
*Lysinibacillus macroides*	C3-53	5.5 ± 0.79^kl^	0.0 ± 0.00^h^	0.0 ± 0.00^m^	31.8 ± 2.62^fgh^
*Bacillus subtilis*	C3-59	34.3 ± 1.58^defg^	20.0 ± 11.55^g^	36.2 ± 0.41^hij^	36.4 ± 2.62^efg^
*Bacillus subtilis*	C3-62	34.3 ± 1.58^defg^	47.0 ± 0.58^bcde^	38.3 ± 1.43^fghi^	56.8 ± 1.31^abc^
*Bacillus subtilis*	C3-72	31.5 ± 1.58^efgh^	50.3 ± 0.33^bcd^	41.2 ± 0.41^defghi^	52.3 ± 3.94^bcd^
Eduarda					
*Paenibacillus taichungensis*	ED2-1	5.9 ± 5.94^kl^	0.0 ± 0.00^h^	0.0 ± 0.00^m^	0.0 ± 0.00^k^
*Bacillus velezensis*	ED2-2	46.6 ± 2.37^abc^	51.0 ± 4.04^bc^	50.6 ± 1.43^abc^	52.3 ± 1.31^bcd^
*Curtobacterium pusillum*	ED2-6	21.0 ± 0.46^hij^	0.0 ± 0.00^h^	0.0 ± 0.00^m^	4.5 ± 2.62^jk^
*Providencia vermicola*	ED3-10	12.8 ± 0.46^jkl^	0.0 ± 0.00^h^	1.6 ± 0.41^m^	4.5 ± 2.62^jk^
*Glutamicibacter mishrai*	ED3-75	11.0 ± 2.37^jkl^	0.0 ± 0.00^h^	26.3 ± 0.41^k^	6.8 ± 1.31^jk^
*Erwinia tasmaniensis*	ED3-79	7.3 ± 0.46^kl^	0.0 ± 0.00^h^	0.0 ± 0.00^m^	37.1 ± 0.76^efg^
*Bacillus subtilis*	ED3-89	41.1 ± 0.79^abcde^	48.3 ± 0.33^bcd^	40.7 ± 2.85^efghi^	55.3 ± 0.76^bcd^
Koala					
*Bacillus pacificus/paranthracis*	KO1-1	26.0 ± 3.16^ghi^	28.0 ± 2.31^fg^	28.8 ± 0.41^jk^	25.0 ± 1.31^ghi^
*Bacillus subtilis*	KO3-11/1	28.8 ± 1.58^fgh^	45.0 ± 0.58^bcde^	37.0 ± 0.71^ghi^	43.2 ± 1.31^def^
*Bacillus subtilis*	KO3-18	39.7 ± 3.16^abcdef^	49.0 ± 1.73^bcd^	42.0 ± 0.71^defghi^	59.8 ± 0.76^abc^
*Pseudomonas oryzihabitans*	KO3-19	15.1 ± 1.58^ijk^	0.0 ± 0.00^h^	22.2 ± 2.14^k^	6.8 ± 1.31^jk^
*Bacillus subtilis*	KO3-26	35.6 ± 0.79^cdefg^	0.0 ± 0.00^h^	4.9 ± 4.99^lm^	54.5 ± 2.62^bcd^
*Mixta theicola*	KO3-44	16.4 ± 0.79^ijk^	0.0 ± 0.00^h^	0.0 ± 0.00^m^	13.6 ± 2.62^ij^
Tibor					
*Priestia aryabhattai*	T1-2	11.0 ± 0.79^jkl^	0.0 ± 0.00^h^	7.4 ± 0.71^lm^	5.3 ± 0.76^jk^
*Bacillus halotolerans*	T2-1	32.9 ± 2.37^defgh^	48.3 ± 0.33^bcd^	38.7 ± 0.41^fghi^	47.7 ± 1.31^cde^
*Bacillus velezensis*	T2-23	**51.1 ± 0.46**^**a**^	49.0 ± 1.73^bcd^	51.9 ± 0.71^ab^	56.8 ± 1.31^abc^
*Arthrobacter oryzae*	T2-25	4.6 ± 2.78^kl^	0.0 ± 0.00^h^	0.0 ± 0.00^m^	3.0 ± 3.03^jk^
*Micrococcus aloeverae*	T2-26	1.8 ± 0.46^l^	0.0 ± 0.00^h^	0.0 ± 0.00^m^	2.3 ± 1.31^jk^
*Bacillus subtilis*	T3-4	38.4 ± 0.79^bcdef^	57.0 ± 0.58^ab^	44.4 ± 0.71^bcdefg^	50.8 ± 0.76^cd^
*Bacillus sonorensis*	T3-5	38.4 ± 2.37^bcdef^	34.0 ± 1.15^ef^	34.6 ± 0.71^ij^	20.5 ± 1.31^hi^
Tajfun					
*Bacillus subtilis*	TF2-1	34.3 ± 1.58^defg^	45.0 ± 2.89^bcde^	48.6 ± 0.41^abcd^	63.6 ± 5.25^ab^
*Bacillus subtilis*	TF3-6	43.8 ± 2.37^abcd^	58.0 ± 1.15^ab^	43.6 ± 0.41^cdefgh^	56.8 ± 3.94^abc^
*Bacillus subtilis*	TF3-7/1	32.0 ± 0.46^defgh^	37.0 ± 1.73^def^	37.0 ± 0.71^ghi^	52.3 ± 3.94^bcd^
*Bacillus subtilis*	TF3-32	42.3 ± 0.33^abcde^	38.0 ± 1.15^cdef^	45.0 ± 0.58^bcdef^	**67.8 ± 0.55**^**a**^

Mean values of percent of inhibition of fungal growth with standard error is shown. Values followed by the same letter within columns are not significantly different (*p* < 0.05), according to Tukey’s HSD test. The values in bold represent the highest percent of inhibition for a particular fungus as a result of the action of a certain isolate

Among them, over 40% of growth inhibitions of all fungal strains were exhibited by *B. amyloliquefaciens* C3-19 (42–68%), *P. polymyxa* C3-36 (49–67%), *B. velezensis* ED2-2 (46–52%), *B. subtilis* ED3-89 (40–55%), *B. velezensis* T2-23 (49–56%), and *B. subtilis* TF3-6 (43–58%). The remaining isolates were active against two or three fungal pathogens, exhibiting moderate to weak activity. *C. beticola* TS4 was the most sensitive fungus, inhibited mostly by *Bacillus* species, recorded with an inhibition rate of over 50%. Among them, the most statistically significant antagonistic activity was achieved by *B. amyloliquefaciens* C3-19 and *B. subtilis* TF3-32 with 68% of inhibition of TS4. Apparently, *F. equiseti* TS2 was the most resistant pathogen to the tested isolates. Moderate to outstanding growth inhibition of this pathogen was detected for antagonistic isolates showing broad range antifungal activity with the highest inhibition rate exhibited by *P. polymyxa* C3-36 (67%). In addition, *P. polymyxa* C3-36 had the most statistically significant inhibition of *F. equiseti* TS2 and *F. oxysporum* TS3, while against *Fusarium* sp. TS1 that significance was achieved by *B. velezensis* T2-23.

In addition, the antagonistic activity of the selected 32 isolates tested against 15 different *Fusarium* strains in an initial screening test. Based on the results obtained, the 10 best strains were selected, and their antagonistic potential was subsequently quantified using the dual culture method (Table 4).

**Table 4:** Antifungal activity (mean ± SE) of selected isolates on *Fusarium* spp. from different origin *in vitro* using a method of dual cultivation

Species	Strain	*F. graminearum* GD1	*F. graminearum* S3-7	*F. graminearum* CIK	*F. oxysporum* S4-2	*F. verticillioides* K67 5.1
*Bacillus amyloliquefaciens*	C3-19	64.8 ± 1.07^ab^	59.0 ± 1.65^ab^	**50.6 ± 2.72**^**a**^	65.1 ± 0.55^b^	62.3 ± 0.00^c^
*Paenibacillus polymyxa*	C3-36	60.2 ± 1.60^abc^	**62.9 ± 4.95**^**a**^	**49.4 ± 0.68**^**a**^	57.5 ± 0.55^cd^	65.1 ± 0.55^abc^
*Bacillus velezensis*	ED2-2	**68.5 ± 1.07**^**a**^	**63.8 ± 2.20**^**a**^	**55.3 ± 0.00**^**a**^	64.2 ± 0.00^b^	66.0 ± 0.00^ab^
*Bacillus velezensis*	T2-23	**66.7 ± 2.14**^**a**^	**66.7 ± 0.55**^**a**^	**54.1 ± 0.68**^**a**^	57.6 ± 1.63^cd^	**67.9 ± 1.09**^**a**^
*Bacillus halotolerans*	C3-16/2.1	54.6 ± 0.54^c^	45.7 ± 1.65^c^	38.8 ± 1.36^bc^	55.7 ± 0.54^de^	57.5 ± 0.55^d^
*Bacillus subtilis*	C3-62	61.1 ± 1.07^abc^	51.4 ± 0.55^bc^	31.8 ± 1.36^c^	56.6 ± 0.00^de^	62.3 ± 0.00^c^
*Bacillus subtilis*	KO3-26	56.5 ± 2.67^bc^	45.7 ± 1.65^c^	36.5 ± 2.72^bc^	61.3 ± 0.54^bc^	55.7 ± 0.54^de^
*Bacillus subtilis*	TF2-1	54.6 ± 0.54^c^	49.5 ± 0.55^bc^	41.2 ± 1.36^b^	**73.6 ± 0.00**^**a**^	63.2 ± 0.55^bc^
*Bacillus subtilis*	TF3-6	53.7 ± 3.21^c^	58.1 ± 1.10^ab^	35.3 ± 0.68^bc^	58.5 ± 1.09^cd^	53.8 ± 0.55^e^
*Bacillus halotolerans*	T2-1	56.5 ± 1.61^bc^	52.4 ± 1.10^bc^	32.9 ± 2.04^c^	52.8 ± 1.09^e^	54.7 ± 1.09^de^
		*F. venatum* IB1I-12	*F. ipomoeae* IB6I-10	*F. foetens* IP27	*F. falciforme* IP31	*F. coffeatum* IP32
*B. amyloliquefaciens*	C3-19	64.9 ± 3.57^ab^	**60.0 ± 2.31**^**a**^	51.4 ± 0.80^abc^	66.7 ± 11.84^ab^	**58.2 ± 8.04**^**a**^
*P. polymyxa*	C3-36	61.9 ± 5.35^abc^	58.0 ± 1.1^5ab^	48.6 ± 2.41^abc^	32.1 ± 5.18^c^	**40.5 ± 2.19**^**a**^
*B. velezensis*	ED2-2	63.9 ± 1.79^abc^	54.0 ± 1.15^abc^	62.5 ± 8.82^ab^	52.6 ± 2.22^bc^	**51.9 ± 2.92**^**a**^
*B. velezensis*	T2-23	**73.2 ± 0.00**^**a**^	**61.0 ± 1.73**^**a**^	47.2 ± 0.00^abc^	46.6 ± 0.43^bc^	**63.3 ± 2.19**^**a**^
*B. halotolerans*	C3-16/2.1	54.6 ± 2.38^bc^	36.0 ± 2.31^g^	41.7 ± 1.60^abc^	50.0 ± 3.70^bc^	**44.3 ± 1.46**^**a**^
*B. subtilis*	C3-62	49.5 ± 1.79^c^	45.0 ± 1.73^def^	**75.0 ± 4.81**^**a**^	42.3 ± 2.22^c^	**67.1 ± 2.92**^**a**^
*B. subtilis*	KO3-26	57.7 ± 2.98^bc^	43.0 ± 1.73^efg^	18.1 ± 0.80^c^	33.3 ± 0.00^c^	**57.0 ± 1.46**^**a**^
*B. subtilis*	TF2-1	57.7 ± 5.36^bc^	51.0 ± 0.58^bcd^	51.4 ± 18.44^abc^	**83.3 ± 2.22**^**a**^	**59.5 ± 7.31**^**a**^
*B. subtilis*	TF3-6	61.9 ± 0.60^abc^	49.0 ± 0.58^cde^	30.6 ± 3.21^bc^	51.3 ± 2.96^bc^	**44.3 ± 4.38**^**a**^
*B. halotolerans*	T2-1	61.9 ± 1.79^abc^	40.0 ± 0.00^fg^	31.9 ± 0.80^bc^	37.2 ± 2.22^c^	**48.1 ± 13.89**^**a**^
		*F. denticulatum* IP39	*F. subglutinans* TS5	*F. nigamai* TS6	*F. semitectum* TS7	*F. solani* TS8
*B. amyloliquefaciens*	C3-19	63.6 ± 0.54^b^	**72.3 ± 9.83**^**a**^	40.5 ± 4.13^b^	**73.7 ± 11.55**^**a**^	**68.6 ± 6.05**^**a**^
*P. polymyxa*	C3-36	57.9 ± 0.54^bc^	54.3 ± 0.61^abcd^	48.4 ± 0.79^ab^	49.5 ± 1.22^bc^	54.3 ± 1.10^abcd^
*B. velezensis*	ED2-2	61.7 ± 0.54^bc^	66.0 ± 4.91^ab^	53.6 ± 2.06^ab^	69.5 ± 0.61^ab^	63.8 ± 0.00^abc^
*B. velezensis*	T2-23	**74.8 ± 0.54**^**a**^	62.8 ± 0.61^abc^	52.4 ± 0.00^ab^	60.0 ± 1.22^abc^	61 ± 1.65^abc^
*B. halotolerans*	C3-16/2.1	59.8 ± 2.7^bc^	44.7 ± 1.23^bcd^	53.6 ± 3.44^ab^	47.4 ± 0.00^bc^	51.4 ± 0.55^bcd^
*B. subtilis*	C3-62	**77.6 ± 2.16**^**a**^	67.0 ± 4.30^ab^	44.1 ± 0.69^b^	63.2 ± 3.04^abc^	42.9 ± 7.70^d^
*B. subtilis*	KO3-26	62.6 ± 1.08^b^	36.2 ± 0.00^d^	36.9 ± 2.06^b^	48.4 ± 0.61^bc^	49.5 ± 0.55^cd^
*B. subtilis*	TF2-1	63.6 ± 1.62^b^	47.9 ± 0.61^bcd^	**64.3 ± 8.25**^**a**^	75.8 ± 7.90^a^	66.7 ± 2.75^ab^
*B. subtilis*	TF3-6	55.1 ± 1.08^c^	61.7 ± 8.60^abc^	50.0 ± 4.12^ab^	50.5 ± 0.61^bc^	43.8 ± 1.65^d^
*B. halotolerans*	T2-1	59.8 ± 0.54^bc^	42.6 ± 1.23^cd^	36.9 ± 2.06^b^	44.2 ± 0.61^c^	41.9 ± 0.55^d^

Mean values of percent of inhibition of fungal growth with standard error is shown. Values followed by the same letter within columns are not significantly different (*p* < 0.05), according to Tukey’s HSD test. The values in bold represent the highest percent of inhibition for a particular fungus as a result of the action of a certain isolate

*F. coffeatum* IP32 was the most sensitive strain showing statistically significant susceptibility to all tested bacteria. Isolate *B. velezensis* T2-23 exhibited the highest statistically significant efficacy in suppressing the proliferation of a greater spectrum of *Fusarium* strains, followed by *B. amyloliquefaciens* C3-19, *B. velezensis* ED2-2, and *B. subtili*s TF2-1. Significantly, the strongest inhibition of all three isolates of *F. graminearum* was achieved equally with *B. velezensis* isolates (ED2-2 and T2-23). In general, the growth of even nine *Fusarium* spp. was successfully inhibited by all tested bacteria with an inhibition rate above 40%.

## Discussion

### Profiling Bacterial Diversity in Sugar Beet Hybrid Seeds by Next-Generation Sequencing Technology

In our study, a metabarcoding approach was used to characterize bacterial diversity in seeds of sugar beet hybrids. Notably, this study represents the pioneering investigation into the bacterial diversity of these hybrids using two distinct methodologies, with the exception of the rhizosphere of the Eduarda hybrid, which has been previously explored [51]. Bacterial community richness and alpha diversity analysis showed that the microbial communities of sugar beet hybrids were more diverse in ED, KO, and T seeds than in C and TF hybrids. PCoA analysis revealed that KO and TF are more distant from the remaining hybrids at the level of genus and ASV. The results of our study indicated that the genotype greatly influences the composition of the bacterial community inhabiting the sugar beet seeds as it was reported for *Rhizoctonia* susceptible and tolerant sugar beet cultivars [22]. The representation of phyla *Actinobacteriota*, *Chloroflexi*, *Firmicutes*, and *Bacteroidetes* in sugar beet seeds of hybrids tested in this study with *Proteobacteria* as the most represented phylum was also reported for sugar beet cultivar H7IM15 [21]. The similarity between cultivar H7IM15 and TF hybrid was reflected in the high presence of the phylum *Firmicutes*. In contrast to the H7IM15 cultivar, *Acidobacteriota* and *Chloroflexi* were detected in ED, KO, and T hybrids. In our study, genera *Pantoea* and *Pseudomonas* were detected in all five hybrids with high RA, with the exception of the low prevalence of *Pseudomonas* in TF. These genera have been highlighted as seed endophytes of different cultivars of sugar beet [21, 52]. *Pantoea* spp. include various life forms such as plant pathogens, plant growth stimulants, and strains used for commercial biocontrol of phytopathogens. Thus, *Pantoea* spp. is a good example of a group that has adapted to a specific niche [22]. Along with *Pantoea* spp. and *Pseudomonas* spp., each hybrid was represented by the dominance of one or two other genera, such as *Kosakonia* (C), *Acinetobacter* (T), and unknown genus from family *Chroococcidiopsaceae* (ED). Wolfgang et al. [22] reported that the abundance of *Kosakonia*, a plant growth–promoting genus, correlates with *Rhizoctonia* tolerance in sugar beet seeds. The observed correlation was in accordance with our result obtained on *Cercospora-*resistant hybrid suggesting that the genus *Kosakonia* plays an important role in sugar beet resistance to the major fungal pathogens of sugar beet. The role of *Acinetobacter* spp. in promoting plant growth, bioremediation and biodegradation was highlighted [53–55]. The TF hybrid was highly dominated by species of the genera *Enterobacter*, *Kosakonia* along with *Pantoea*. These taxa all belong to the *Enterobacterales* order, highlighting a taxonomic similarity among the dominant members of the bacterial community. The codominance of these genera suggests potential ecological interactions and niche specialization within this bacterial order. There are many PGP characteristics that *Enterobacter* spp. are known to possess, including the capability to fix nitrogen; solubilize phosphorus in soil; produce antibiotics; secrete siderophore products, chitinase, ACC deaminase, and hydrolytic enzymes in addition to exopolysaccharides; and increase soil porosity [56]. These properties are expressed by a variety of *Enterobacter* strains and contribute to plant growth and control of soil-borne plant diseases [57]. Other genera apart of *Acinetobacter*, *Enterobacter*, *Enterococcus*, and *Kosakonia*, detected in all hybrids, were *Glutamicibacter* and *Marinilactibacillus*, although their abundance depended on the hybrids. Representatives of these genera are associated with PGP traits [22, 56, 58–61], with most species classified as psychrotolerant/halotolerant bacteria [58–61]. In previous studies, *Weissella* spp. were reported as an endophyte of sugar beet seeds [21], and its high abundance in TF hybrids was the main characteristic that separated it from other analyzed hybrids. Hybrid-specific genera such as *Apibacter* (ED), *Alloscardovia* (KO), and *Pleurocapsa* (T) are mentioned as components of the marine, human, and animal microbiome [62–65], while their occurrence in the bacteriome of plants and in PGP capabilities is not yet known. The hybrid-specific genera *Arthrobacter* (T) and *Klebsiella* (TF) are known for their potential to promote plant growth [66, 67].

###  Diversity of Culturable Bacteria from Rhizosphere, Roots, and Seeds of Sugar Beet Hybrids

Within the field of biology, the concept of phylosymbiosis suggests that closely related species tend to display greater similarity in their microbiomes in comparison to distantly related species [68]. This aligns with research showing that plant genotypes, cultivars, developmental cycles, and other factors strongly influence endophytic microbial diversity and community structure [21]. Studying the microbiota of hybrid plants can reveal insights into the phylosymbiotic patterns within the plant family. The bacterial diversity present in the rhizosphere, roots, and seeds of five sugar beet hybrids, known as Eduarda, Koala, Tibor, Tajfun, and *Cercospora*-resistant, was thoroughly examined. Analysis of culturable bacteria showed that rhizospheres of all hybrids shared the common species *L. macroides*, while there were also species similarities between individual hybrids. The presence of *Lysinibacillus* strains in the rhizosphere has several benefits for the host plant, such as preventing cadmium uptake in plants [69] and attenuating the virulence of the pathogenic species *Pectobacterium carotovorum* by degrading the AHL signal and interrupting the pathogen's quorum sensing [70]. The rhizosphere is a region of rich, mostly soil derived, microbial diversity, influenced by plant roots through rhizodeposition of exudates, mucilage, and sloughed cells [12]. Considering that the composition of root exudates is influenced by the plant genome [4], as has been shown for the rhizosphere microbiome of *Arabidopsis thaliana* [12], the different genotypes of these five hybrids could be a determinant of bacterial diversity, as all hybrids had at least one hybrid-specific species in their rhizospheres. In the study by Zachow et al. [20] investigating differences in the rhizosphere microbiome of wild sugar beet (*Beta vulgaris* ssp. *maritima*) and modern varieties, it was found that greater bacterial diversity was associated with wild beet. Moreover, the same study revealed that they shared similarities in *Pseudomonas* and *Stenotrophomonas* species. The rhizosphere of the sugar beet was earlier showed to be colonized with *B*. *subtilis*, *B*. *halotolerans*, *B*. *amyloliquefaciens*, and *Bacillus* s*afensis* [13], while the first three species were associated with the roots and seeds of investigated hybrids. Although *Bacillus* is ubiquitous in various environments, its abundance in cultivation can be easily overestimated due to copiotrophy and endospore formation. To the best of our knowledge, this is the first study to investigate the diversity of culturable bacteria in sugar beet seeds. Previous research also indicated a high diversity of bacterial taxa within the roots of sugar beet [71, 72]. Besides the genus *Bacillus*, species from genera *Arthrobacter*, *Micrococcus*, *Microbacterium*, *Curtobacterium*, *Rhodococcus*, and *Staphylococcus* were previously reported as root endophytes [71], which was in accordance with our study.

### Sugar Beet–Associated Cultured Endophytic (Seed and Roots) and Rhizospheric Bacteria with Plant Growth–Promoting Properties

Plants harbor diverse bacterial communities whose interactions determine plant health and productivity. Determining whether bacteria promote or inhibit plant growth is an important step in the study to develop a PGPB-based inoculant. Moreover, indigenous bacterial strains are more suitable as growth promoters than new strains introduced from another location because plant-microbe interaction depends on different environmental parameters and indigenous PGPB are highly adapted to habitat [73]. Today, to meet the nutritional needs of plants and protect them from phytopathogens, large amounts of chemical fertilizers and pesticides are increasingly used [8]. However, it is well known that their excessive use leads to groundwater and soil pollution and a growing problem of resistance to pathogens. Since the use of chemicals in agriculture can never be completely eliminated, there is an increasing reliance on beneficial bacteria to alleviate this problem [74]. To investigate PGP abilities of culturable collection of bacteria isolated from five sugar beet hybrids, a panel of tests was performed. All 32 selected sugar beet isolates exhibited the ability to fix nitrogen to varying degrees. This is very important from the applicative standpoint, because the lack of available nitrogen in the soil on which the plant grows is one of the main factors that limit its development [75]. The majority of the soluble inorganic phosphorus used as chemical fertilizer is also immobilized quickly after application, rendering it unavailable to plants and resulting in its ineffectiveness [7]. As a result, one of the key characteristics of PGPB is the solubilization and mineralization of phosphorus by phosphate-solubilizing bacteria. In sugar beet isolates, the vast majority possessed this ability. *P. vermicola* ED3-10 and *M. theicola* KO3-44 exhibited excellent IAA production which is in accordance with the previous reports [76, 77]. IAA synthesized by bacteria increases root surface area and length, giving the plant better access to soil-derived nutrients. *M. theicola* has been reported with the significant improvement of root elongation in *Zea mays* related to IAA, in addition to many other stimulatory performances to this host [76]. In addition, bacterial IAA loosens plant cell walls, facilitating increased root exudation that provides additional nutrients to support the growth of rhizosphere bacteria [7]. Both *P*. *vermicola* and *M*. *theicola* showed to be great candidates for stimulation of plant growth as they both shared other PGP characteristics such as nitrogen fixation, phosphate solubilization, siderophore production, motility, and salinity tolerance up to 8% of NaCl. Also, *M*. *theicola* proved to be an excellent producer of EPS. Still, the production of hydrolytic enzymes was not recorded for these two isolates. For *P. vermicola*, a wide range of promoting characteristics for plant growth has been shown, of which siderophore production, phosphate solubilization, and nitrogen fixation [77–79] were also confirmed in our study. Furthermore, its biocontrol potential towards phytopathogenic soil-borne fungi and nematodes was reported [77]. Previous studies have demonstrated the efficacy of PGP bacteria in stimulating overall plant growth of sugar beet, including enzyme activity, sugar content, fiber and storage root development, and leaf yield, as well as leaf chlorophyll and nutrient content under drought stress [80]. Considering that iron deficiency leads to disruption of plant nutrition [73], the excellent siderophore production observed in this study, especially for the isolates of *B. subtilis* and *B. velezensis*, is a beneficial trait that could increase iron uptake by the host plant. These PGPB can also act as biocontrol agents through competition for iron as micronutrient, since their siderophores have a much greater affinity for iron than the fungal pathogens, making them unavailable [7].The ability of PGP’s bacteria to produce HCN, which is crucial for protecting crop plants from disease, is another important feature [48]. The known effects of HCN include disruption of the energy supply, inhibition of electron transport, and cell death [81]. The antifungal activity of *B. halotolerans* C3-16/2.1, *B. zhangzhouensis* C3-50, *B. subtilis* C3-72, and *B. subtilis* T3-4, apart from the likely existence of antifungal secondary metabolites, might be related to HCN synthesis, as well since these strains exhibited strong HCN production and suppression of fungal pathogens in this study. Suppression of *Fusarium oxysporum* f. sp. *lycopersici* spores has been previously reported for HCN produced by *Pseudomonas fluorescens* [82].

### Sugar Beet–Associated Cultured Endophytic (Seed and Roots) and Rhizospheric Bacteria with Exoenzymatic Activity

In addition, PGPBs are known to protect host plants from phytopathogens via other mechanisms, including the production of hydrolytic enzymes [8]. Sugar beet isolates exhibited various enzymatic activities involving amylase, protease, lipase, xylanase, mannanase, cellulase, gelatinase, and pectinase production. Gelatinases which were often associated with biocontrol of plant pathogens such as insects and nematodes [83], were the most commonly produced enzymes in this study. Furthermore, the importance of gelatinases in disease protection of rice plants against spore-dispersing pathogenic fungi has been highlighted [83]. In another view regarding benefits to the plant, microorganisms with ability of producing cellulases, pectinases, xylanases, and amylases play an important role in the decomposition of organic matter and nutrient mineralization. In this study, *B. halotolerans* C3-16/2.1 and strains of *B. velezensis* (T2-23, ED2-2), *B. subtilis* T3-4, and *P. polymyxa* C3-36 showed the best exoenzymatic activity, while in some cases, the production of lipases, xylanases, and proteinases was absent. The enzymes such as cellulose, pectinase, xylanase, and protease are responsible for hydrolytic processes that allow endophytes to colonize plant tissues and establish a symbiotic relationship between endophytes and host plants [84]. The production of these enzymes was found in almost all *Bacillus* and *Paenibacillus* isolates from sugar beet, which was expected due to the fact that most were endophytes. In addition, the cellulolytic, lipolytic, and proteolytic activities of endophytes are known to protect host plants from pathogenic microorganisms by cell wall lysis of pathogens [7].

### Sugar Beet–Associated Cultured Endophytic (Seed and Roots) and Rhizospheric Bacteria Tolerate Abiotic Stress

Salinity, flooding, drought, cold, and heavy metals are a few examples of the various abiotic stressors to which plants are exposed [85]. Crops exposed to these stressors can suffer significant production losses. Soil salinization is believed to have negative impacts on over 20% of all agricultural land worldwide. High soil salinity, primarily caused by the presence of NaCl, negatively impacts the growth and yield of most crops due to their limited salt tolerance. This is because the elevated salt levels interfere with the plants’ hormonal and nutritional processes, leading to reduced productivity and hindered growth [85]. Tolerance of sugar beet isolates to salt stress was evident as they grew successfully at elevated salt concentrations in the medium. *B. halotolerans* C3-16/2.1 proved to be an excellent candidate for stimulating plant growth in saline environments, showing the greatest tolerance to salinity, even at 10% NaCl. *B. halotolerans* was reported to protect plant growth and alleviate salt stress in wheat by modulating phytohormone synthesis and regulating osmotic balance, ion homeostasis, and gene expression [85]. Another important abiotic stress which leads to negative impact on crop production is agricultural drought as a period of soil moisture deficit resulting from a combination of rainfall deficiency and excessive evapotranspiration in a particular region [9]. Twenty-one isolates from sugar beets could be classified as drought-tolerant bacteria based on their ability to grow under high moisture deficit stress conditions. *B. subtilis* C3-62 and TF2-1 grew efficiently at the maximum concentration of PEG with no visible reduction in growth compared to the control and therefore could be used as stress-tolerant PGPR in soils facing drought. Bacteria can survive under stress conditions due to the production of EPS, which protects microorganisms from water stress by enhancing water retention and regulating the diffusion of organic carbon sources [49]. Since both *B. subtilis* strains were EPS-producing bacteria, this could be a reason for their high drought tolerance. EPS also helps microorganisms to irreversibly attach to and colonize the root, as it forms a network of fibrillar material that permanently connects the bacteria to the root surface [49], which is another important factor that favors these two strains from an application point of view. Regulation of plant hormone levels, especially auxin and ethylene, is perhaps the most important mechanism PGPB uses to promote plant development, especially in the presence of environmental conditions such as drought [86]. The amount of ACC in stressed plants is reduced by ACC deaminase–containing PGPR, which in turn reduces the amount of stress-induced ethylene production and subsequently plant damage. Since plants are frequently exposed to stress factors that trigger the synthesis of ethylene [49], the ACC deaminase–producing bacteria *P. aryabhattai* T1-2, *B. subtilis* KO3-18, and *C. pusillum* ED2-6 could be good candidates for the production of bioinoculants to control abiotic stress in plants.

### Sugar Beet–Associated Cultured Endophytic (Seed and Roots) and Rhizospheric Bacteria as Potential Biocontrol Agents Based on Antifungal Potential *In Vitro*

The antagonistic activity of selected isolates was evaluated against several fungal pathogens known to cause *Fusarium* wilt [16], root rot [18], and *Cercospora* leaf spot disease [15] in sugar beet plants, as well as against other potential pathogens of *Fusarium* spp. [57, 87, 88]. Growth of fungal pathogens isolated from sugar beet was highly suppressed by *P. polymyxa*, *B. subtilis*, *B. halotolerans*, and *B. velezensis* strains. The great antagonistic potential of different *Bacillus* strains from the sugar beet rhizosphere against the most devastating pathogen *C. beticola* has been reported with the greatest activity achieved with *Bacillus* spp. strains (60-71% depending on the strain) [89]. Among 12 strains of *B. subtilis* tested, the best antifungal candidate against *C. beticola* was TF2-1. *B. halotolerans*, *B. velezensis*, *B. subtilis*, and *P. polymyxa* were the most successful in inhibiting growth of *F. equiseti*, where *P. polymyxa* C3-36 had the most statistically significant inhibition. Also, *P. polymyxa* had the statistically greatest activity against *F. oxysporum*. Inhibition of mycelial growth of *Cercospora* spp. and *F. oxysporum* [90, 91] has been reported for *B. velezensis*. The antifungal activity of *B. velezensis* is related to the production of secondary metabolites and hydrolytic enzymes [91], with bacillomycin D being recognized as the main compound potentially responsible for the broad antifungal activity [92]. In addition, isolate *P. polymyxa* C3-36 had excellent antifungal activity against all *Fusarium* strains tested, which already confirmed for various strains of *P. polymyxa* against *F. oxysporum* f. sp. *lycopersici* [93]. Both strains of *B. halotolerans* in our study were able to suppress the growth of each pathogen tested. Strains of *B. halotolerans* were already linked to the antifungal activity against various plant pathogens, including species of *Fusarium*, *Rhizoctonia*, and *Botrytis* genera [94, 95]. All tested strains of *F. graminearum*, which is known as a causative agent of head blight disease of cereal crops and sugar beet [16, 96], were highly sensitive to our *B. subtilis*, *B. halotolerans*, *B. velezensis*, and *P. polymyxa* strains. In general, the isolates of *B. velezensis* and *P. polymyxa* showed excellent antagonistic potential against all fungal pathogens tested.

## Conclusion

The present study provides an interesting insight into the composition of seed-borne endophytes in five different sugar beet hybrids. The differences in the occurrence and abundance of certain genera in hybrid seeds as well as the presence of unique species clearly indicate that plant genotypes contribute to the structure of the seed-associated bacterial community of sugar beet. The prevalence of *Pantoea* and *Pseudomonas* species in the seeds of sugar beet hybrids was strikingly high, with the exception of the TF hybrid, which had a distinct profile characterized by a considerable presence of *Weissella* sp. Furthermore, the abundance of the *Kosakonia* genus in the *Cercospora*-resistant hybrid potentially contributes significantly to improved sugar beet resistance, which is consistent with its occurrence in other fungus-resistant sugar beet hybrids. This is also underlined by the fact that isolates with robust antifungal properties were predominantly derived from this particular hybrid. Furthermore, the hybrid genetic influence on the composition of the cultured rhizosphere and root endophyte community is visible through the diversity of isolates obtained isolates from different hybrids. The present study shows that the seed and root endophytes, as well as rhizospheric bacterial strains exhibit a broad spectrum of activities and thus offer a high potential for the development of plant probiotics and biological control agents. Based on their investigated activities, the obtained endophytes can be described as drought and halotolerant, phosphate solubilizing bacteria as well as bioproducers of siderophores, exopolysaccharides, indole-3-acetic acid, gelatinases, and amylases. Further studies will focus on testing the compatibility of the best candidates, forming viable “bottom-up” consortia, and monitoring their effects on sugar beet development and growth and pathogen resistance under controlled conditions and in the field.

### Supplementary Information

ESM 1 (DOCX 107 kb)

## Data Availability

The data presented in this study are openly available as BioProject ID: PRJNA954324 in the NCBI repository (https://www.ncbi.nlm.nih.gov/bioproject/954324).
