# Supplementary material for: Insights into Endophytic and Rhizospheric Bacteria of Five Sugar Beet Hybrids in Terms of Their Diversity, Plant-Growth Promoting, and Biocontrol Properties
Source: Microb Ecol. 2023 Dec 27;87:19. doi: 10.1007/s00248-023-02329-0 (PMC10751262; doi:10.1007/s00248-023-02329-0)
Supplement: Supplementary file 1 [file 248_2023_2329_MOESM1_ESM.docx]

**Supplementary Information**

**Insights into endophytic and rhizospheric bacteria of five sugar beet hybrids in terms of their diversity, plant-growth promoting and biocontrol properties**

**Marija Petrović^1^, Tamara Janakiev^1^, Milica Ljaljević Grbić^1^, Nikola Unković^1^, Tatjana Stević^2^, Slavoljub Vukićević^3^, Ivica Dimkić^1^**

^1^University of Belgrade – Faculty of Biology, Studentski trg 16, 11158 Belgrade, Serbia

^2^Institute for Medicinal Plant Research “Dr Josif Pančić”, Tadeuša Košćuška 1, 11000 Belgrade, Serbia

^3^FERTICO DOO, Vojvode Putnika 94a, 22320 Indjija, Serbia

***Correspondence to:**

Ivica Dimkić, PhD

Associate Research Professor

Chair of Biochemistry and Molecular Biology

University of Belgrade - Faculty of Biology

Studentski trg 16

11158 Belgrade, Serbia

E-mail: ivicad@bio.bg.ac.rs

orcid.org/0000-0002-0425-5938

**Table S1. Origin of fungal strains used in a dual culture test.**

| **Strain** | **Origin** |
| --- | --- |
| *Fusarium graminearum* GD1 | Soybean |
| *Fusarium graminearum* S3-7 | Soil |
| *Fusarium graminearum* CIK | Soil |
| *Fusarium oxysporum* S4-2 | Soil |
| *Fusarium verticilioides* K675.1 | Soil |
| *Fusarium venatum* IB1I-12 | Olive |
| *Fusarium ipomoeae* IB6I-10 | Olive |
| *Fusarium foetens* IP27 | Tomato |
| *Fusarium coffeatum* IP32 | Pepper |
| *Fusarium falciforme* IP31 | Pepper |
| *Fusarium denticulatum* IP39 | Zucchini |
| *Fusarium* sp. TS1 | Sugar beet |
| *Fusarium equseti* TS2 | Sugar beet |
| *Fusarium oxysporum* TS3 | Sugar beet |
| *Cercospora beticola* TS4 | Sugar beet |
| *Fusarium subglutinans* TS5 | Sugar beet |
| *Fusarium nigamai* TS6 | Sugar beet |
| *Fusarium semitectum* TS7 | Maize |
| *Fusarium solani* TS8 | Sugar beet |

**Table S2.** The number of unique species of five sugar beet hybrids from the rhizosphere, roots, and seeds.

| **All hybrids** | **No. of species** | **No. of unique species** |
| --- | --- | --- |
| **Rhizosphere** | | |
| *Cercospora-*resistant | 4 | 4 |
| Eduarda | 4 | 4 |
| Koala | 7 | 5 |
| Tajfun | 4 | 4 |
| Tibor | 7 | 5 |
| Overall number | **26** | **22** |
| **Root endophytes** | | |
| *Cercospora-*resistant | 5 | 3 |
| Eduarda | 7 | 6 |
| Koala | 5 | 3 |
| Tajfun | 2 | 2 |
| Tibor | 18 | 17 |
| Overall number | **37** | **31** |
| **Seed endophytes** | | |
| *Cercospora-*resistant | 25 | 16 |
| Eduarda | 26 | 20 |
| Koala | 28 | 17 |
| Tajfun | 9 | 7 |
| Tibor | 5 | 3 |
| Overall number | **93** | **63** |

**Table S3. Identification of culturable bacteria according to the sequencing of 16S rRNA gene.**

| **Strain** | **Species** | **Max Score** | **Total Score** | **Query Cover** | **Per. of ident.** |
| --- | --- | --- | --- | --- | --- |
| **Eduarda hybrid** | | | | | |
| **Rhizosphere** | | | | | |
| ED1-1 | *Lysinibacillus macroides* | 1567 | 1567 | 100% | 99.31% |
| ED1-2 | *Cytobacillus ciccensis* | 1624 | 1624 | 100% | 99.77% |
| ED1-3 | *Bacillus badius* | 1605 | 1605 | 100% | 99.89% |
| ED1-4 | *Lysinibacillus fusiformis* | 1611 | 1611 | 100% | 100.00% |
| **Root endophytes** | | | | | |
| ED2-1 | *Paenibacillus taichungensis* | 1596 | 1596 | 100% | 99.43% |
| ED2-2 | *Bacillus velezensis* | 1616 | 1616 | 100% | 99.89% |
| ED2-4 | *Knoellia locipacati* | 1498 | 1498 | 100% | 98.70% |
| ED2-6 | *Curtobacterium pusillum* | 1434 | 1434 | 100% | 99.62% |
| ED2-7 | *Curtobacterium pusillum* | 1399 | 1399 | 100% | 99.48% |
| ED2-8 | *Microbacterium thalassium* | 1500 | 1500 | 100% | 98.59% |
| ED2-10 | *Mycetocola manganoxydans* | 1493 | 1493 | 100% | 99.75% |
| **Seed endophytes** | | | | | |
| ED3-1 | *Staphylococcus succinus* subsp. *succinus* | 1615 | 1615 | 100% | 100.00% |
| ED3-2 | *Pseudomonas oryzihabitans* | 1528 | 1528 | 100% | 98.95% |
| ED3-3 | *Bacillus mobilis* | 1644 | 1644 | 100% | 100.00% |
| ED3-4 | *Kosakonia cowanii* | 1557 | 1557 | 100% | 99.76% |
| ED3-10 | *Providencia vermicola* | 1567 | 1567 | 100% | 99.42% |
| ED3-13 | *Acinetobacter lactucae* | 1594 | 1594 | 100% | 100.00% |
| ED3-21 | *Pseudomonas oryzihabitans* | 1533 | 1533 | 100% | 98.84% |
| ED3-42 | *Pseudomonas oryzihabitans* | 1533 | 1533 | 100% | 98.84% |
| ED3-43 | *Microbacterium testaceum* | 1544 | 1544 | 100% | 99.76% |
| ED3-46 | *Acinetobacter lactucae* | 1594 | 1594 | 100% | 100.00% |
| ED3-48 | *Providencia sneebia* | 1533 | 1533 | 100% | 98.73% |
| ED3-48/1 | *Klebsiella michiganensis* | 1607 | 1607 | 100% | 99.54% |
| ED3-74 | *Bacillus zhangzhouensis* | 1607 | 1607 | 100% | 99.89% |
| ED3-74/1 | *Erwinia persicina* | 1585 | 1585 | 100% | 100.00% |
| ED3-75 | *Glutamicibacter mishrai* | 1507 | 1507 | 100% | 99.88% |
| ED3-76 | *Sanguibacter keddieii* | 1552 | 1552 | 100% | 99.76% |
| ED3-77 | *Micrococcus luteus* | 1543 | 1543 | 100% | 99.64% |
| ED3-78 | *Erwinia tasmaniensis* | 1548 | 1548 | 100% | 98.96% |
| ED3-79 | *Erwinia tasmaniensis* | 1548 | 1548 | 100% | 98.96% |
| ED3-81 | *Curtobacterium pusillum* | 1539 | 1539 | 100% | 99.53% |
| ED3-82 | *Erwinia persicina* | 1585 | 1585 | 100% | 100.00% |
| ED3-83 | *Micrococcus aloeverae* | 1550 | 1550 | 100% | 99.88% |
| ED3-84 | *Pseudomonas oryzihabitans* | 1533 | 1533 | 100% | 98.84% |
| ED3-85 | *Alkalihalobacillus rhizosphaerae* | 1408 | 1408 | 100% | 99.11% |
| ED3-86 | *Frigoribacterium faeni* | 1400 | 1400 | 100% | 99.35% |
| ED3-89 | *Bacillus subtilis* | 1635 | 1635 | 100% | 99.89% |
| **Tibor hybrid** | | | | | |
| **Rhizosphere** | | | | | |
| T1-1 | *Lysinibacillus macroides* | 1580 | 1580 | 100% | 99.42% |
| T1-2 | *Priestia aryabhattai* | 1616 | 1616 | 100% | 100.00% |
| T1-3 | *Bacillus wiedmannii/proteolyticus/fungorum* | 1646 | 1646 | 100% | 99.89% |
| T1-4 | *Lysinibacillus macroides* | 1580 | 1580 | 100% | 99.42% |
| T1-5 | *Bacillus altitudinis/aerophilus/stratosphericus* | 1607 | 1607 | 100% | 100.00% |
| T1-6 | *Bacillus altitudinis/aerophilus/stratosphericus* | 1607 | 1607 | 100% | 100.00% |
| T1-6/1 | *Lysinibacillus pakistanensis* | 1628 | 1628 | 100% | 100.00% |
| **Root endophytes** | | | | | |
| T2-1 | *Bacillus halotolerans* | 1615 | 1615 | 100% | 100.00% |
| T2-2 | *Paenibacillus azotifigens* | 1491 | 1491 | 100% | 97.59% |
| T2-6 | *Neobacillus niacini* | 1557 | 1557 | 100% | 98.85% |
| T2-8 | *Neorhizobium huautlense* | 1471 | 1471 | 100% | 99.50% |
| T2-9 | *Micrococcus aloeverae* | 1550 | 1550 | 100% | 99.76% |
| T2-14 | *Kocuria rosea* | 1552 | 1552 | 100% | 99.76% |
| T2-15 | *Paenibacillus catalpae/lupini* | 1581 | 1581 | 100% | 99.54% |
| T2-16 | *Rhodococcus corynebacterioides* | 1546 | 1546 | 100% | 99.88% |
| T2-18 | *Rathayibacter tritici* | 1539 | 1539 | 100% | 99.64% |
| T2-19 | *Microbacterium testaceum* | 1493 | 1493 | 100% | 98.81% |
| T2-20 | *Paenibacillus lautus* | 1596 | 1596 | 100% | 99.77% |
| T2-21 | *Rhodococcus cerastii* | 1249 | 1249 | 100% | 99.85% |
| T2-22 | *Micrococcus luteus* | 1555 | 1555 | 100% | 99.65% |
| T2-23 | *Bacillus velezensis* | 1620 | 1620 | 100% | 99.89% |
| T2-25 | *Arthrobacter oryzae* | 1537 | 1537 | 100% | 99.53% |
| T2-26 | *Micrococcus aloeverae* | 1561 | 1561 | 100% | 100.00% |
| T2-28 | *Metabacillus indicus* | 1506 | 1506 | 100% | 97.93% |
| T2-32 | *Massilia timonae* | 1583 | 1583 | 100% | 99.88% |
| **Seed endophytes** | | | | | |
| T3-3 | *Bacillus sonorensis* | 1602 | 1602 | 100% | 100.00% |
| T3-4 | *Bacillus subtilis* | 1615 | 1615 | 100% | 100.00% |
| T3-5 | *Bacillus sonorensis* | 1602 | 1602 | 100% | 100.00% |
| T3-6 | *Bacillus sonorensis* | 1602 | 1602 | 100% | 100.00% |
| T3-6/1 | *Acinetobacter lactucae* | 1594 | 1594 | 100% | 100.00% |
| **Tajfun hybrid** | | | | | |
| **Rhizosphere** | | | | | |
| TF1-1 | *Lysinibacillus pakistanensis* | 1616 | 1616 | 100% | 100.00% |
| TF1-2 | *Bacillus mobilis* | 1615 | 1615 | 100% | 100.00% |
| TF1-3 | *Lysinibacillus fusiformis* | 1605 | 1605 | 100% | 100.00% |
| TF1-4 | *Lysinibacillus macroides* | 1585 | 1585 | 100% | 99.43% |
| **Root endophytes** | | | | | |
| TF2-1 | *Bacillus subtilis* | 1620 | 1620 | 100% | 100.00% |
| TF2-6 | *Micrococcus luteus/aloeverae* | 1482 | 1482 | 100% | 98.34% |
| **Seed endophytes** | | | | | |
| TF3-6 | *Bacillus subtilis* | 1615 | 1615 | 100% | 100.00% |
| TF3-7/1 | *Bacillus subtilis* | 1615 | 1615 | 100% | 100.00% |
| TF3-16 | *Bacillus licheniformis* | 1602 | 1602 | 100% | 99.77% |
| TF3-20 | *Microbacterium paludicola* | 1517 | 1517 | 100% | 99.05% |
| TF3-27 | *Paenibacillus lautus* | 1604 | 1604 | 100% | 99.89% |
| TF3-29 | *Weizmannia ginsengihumi* | 1613 | 1613 | 100% | 100.00% |
| TF3-32 | *Bacillus subtilis* | 1615 | 1615 | 100% | 100.00% |
| TF3-33 | *Brevundimonas nasdae/vesicularis* | 1452 | 1452 | 100% | 99.38% |
| TF3-82 | *Bacillus oleivorans* | 1511 | 1511 | 100% | 99.05% |
| **Koala hybrid** | | | | | |
| **Rhizosphere** | | | | | |
| KO1-1 | *Bacillus pacificus/paranthracis* | 1615 | 1615 | 100% | 100.00% |
| KO1-2 | *Rossellomorea marisflavi* | 1604 | 1604 | 100% | 100.00% |
| KO1-3 | *Lysinibacillus macroides* | 1574 | 1574 | 100% | 99.42% |
| KO1-3/2 | *Rossellomorea marisflavi* | 1604 | 1604 | 100% | 100.00% |
| KO1-4 | *Bacillus wiedmannii/proteolyticus/fungorum* | 1615 | 1615 | 100% | 100.00% |
| KO1-5 | *Lysinibacillus macroides* | 1574 | 1574 | 100% | 99.42% |
| KO1-7 | *Brevibacillus reuszeri* | 1580 | 1580 | 100% | 99.42% |
| **Root endophytes** | | | | | |
| KO2-1 | *Micrococcus aloeverae* | 1559 | 1559 | 100% | 100.00% |
| KO2-2 | *Micrococcus aloeverae* | 1548 | 1548 | 100% | 99.76% |
| KO2-3 | *Micrococcus terreus* | 1561 | 1561 | 100% | 99.88% |
| KO2-4 | *Micrococcus aloeverae* | 1559 | 1559 | 100% | 100.00% |
| KO2-6 | *Staphylococcus epidermidis* | 1607 | 1607 | 100% | 100.00% |
| **Seed endophytes** | | | | | |
| KO3-2 | *Kosakonia cowanii* | 1430 | 1430 | 100% | 99.87% |
| KO3-3 | *Bacillus haynesii* | 1598 | 1598 | 100% | 99.54% |
| KO3-4 | *Micrococcus aloeverae* | 1559 | 1559 | 100% | 100.00% |
| KO3-9 | *Mixta theicola* | 1384 | 1384 | 100% | 98.23% |
| KO3-10 | *Alkalihalobacillus gibsonii* | 1587 | 1587 | 100% | 99.31% |
| KO3-11/1 | *Bacillus subtilis* | 1620 | 1620 | 100% | 100.00% |
| KO3-12 | *Bacillus zhangzhouensis* | 1607 | 1607 | 100% | 99.89% |
| KO3-14 | *Enterococcus gallinarum* | 1628 | 1628 | 100% | 99.89% |
| KO3-17 | *Pseudomonas oryzihabitans* | 1539 | 1539 | 100% | 99.18% |
| KO3-18 | *Bacillus subtilis* | 1620 | 1620 | 100% | 100.00% |
| KO3-19 | *Pseudomonas oryzihabitans* | 1474 | 1474 | 100% | 98.79% |
| KO3-20 | *Curtobacterium pusillum* | 1539 | 1539 | 100% | 99.53% |
| KO3-26 | *Bacillus subtilis* | 1620 | 1620 | 100% | 100.00% |
| KO3-28 | *Pantoea agglomerans/Curtobacterium plantarum* | 1483 | 1483 | 100% | 99.27% |
| KO3-28/1 | *Pantoea agglomerans* | 1541 | 1541 | 100% | 99.07% |
| KO3-30 | *Pantoea allii* | 1504 | 1504 | 100% | 97.93% |
| KO3-32 | *Pseudomonas oryzihabitans* | 1533 | 1533 | 100% | 99.07% |
| KO3-36 | *Okibacterium fritillariae* | 1550 | 1550 | 100% | 99.65% |
| KO3-38 | *Bacillus haynesii* | 1592 | 1592 | 100% | 99.43% |
| KO3-39 | *Sanguibacter inulinus* | 1537 | 1537 | 100% | 99.76% |
| KO3-44 | *Mixta theicola* | 1384 | 1384 | 100% | 98.23% |
| KO3-46 | *Microbacterium arborescens/imperiale* | 1476 | 1476 | 100% | 98.91% |
| KO3-47 | *Curtobacterium pusillum* | 1539 | 1539 | 100% | 99.53% |
| KO3-48 | *Bacillus zhangzhouensis* | 1607 | 1607 | 99% | 100.00% |
| KO3-48/1 | *Bacillus zhangzhouensis* | 1613 | 1613 | 100% | 100.00% |
| KO3-49 | *Microbacterium saccharophilum* | 1474 | 1474 | 100% | 98.79% |
| KO3-51 | *Curtobacterium pusillum* | 1506 | 1506 | 100% | 98.82% |
| KO3-80 | *Curtobacterium pusillum* | 1544 | 1544 | 100% | 99.64% |
| ***Cercospora*-resistant hybrid** | | | | | |
| **Rhizosphere** | | | | | |
| C1-1 | *Bacillus toyonensis/thuringiensis* | 1618 | 1618 | 100% | 100.00% |
| C1-2 | *Bacillus pacificus/paranthracis* | 1555 | 1555 | 100% | 100.00% |
| C1-3 | *Lysinibacillus macroides* | 1574 | 1574 | 100% | 99.31% |
| C1-5 | *Brevibacillus borstelensis* | 1591 | 1591 | 100% | 99.66% |
| **Root endophytes** | | | | | |
| C2-1 | *Bacillus pseudomycoides* | 1607 | 1607 | 100% | 100.00% |
| C2-5 | *Micrococcus luteus* | 1548 | 1548 | 100% | 99.65% |
| C2-6 | *Micrococcus luteus* | 1548 | 1548 | 100% | 99.65% |
| C2-7 | *Massilia timonae* | 1581 | 1581 | 100% | 99.88% |
| C2-7/1 | *Massilia timonae* | 1581 | 1581 | 100% | 99.88% |
| **Seed endophytes** | | | | | |
| C3-13 | *Corynebacterium doosanense* | 1533 | 1533 | 100% | 99.76% |
| C3-16/2 | *Bacillus mojavensis* | 1622 | 1622 | 100% | 99.89% |
| C3-16/2.1 | *Bacillus halotolerans* | 1635 | 1635 | 100% | 100.00% |
| C3-16/2.3 | *Bacillus halotolerans* | 1635 | 1635 | 100% | 100.00% |
| C3-17 | *Curtobacterium pusillum* | 1541 | 1541 | 100% | 99.53% |
| C3-18 | *Pseudoclavibacter helvolus* | 1539 | 1539 | 100% | 99.76% |
| C3-19 | *Bacillus amyloliquefaciens* | 1594 | 1594 | 100% | 99.88% |
| C3-36 | *Paenibacillus polymyxa* | 1618 | 1618 | 100% | 100.00% |
| C3-40 | *Bacillus subtilis* | 1615 | 1615 | 100% | 100.00% |
| C3-44 | *Pantoea agglomerans/Curtobacterium plantarum* | 1487 | 1487 | 100% | 99.27% |
| C3-47 | *Bacillus altitudinis/aerophilus/stratosphericus* | 1613 | 1613 | 100% | 100.00% |
| C3-50 | *Bacillus zhangzhouensis* | 1616 | 1616 | 100% | 100.00% |
| C3-50/1 | *Bacillus zhangzhouensis* | 1607 | 1607 | 100% | 100.00% |
| C3-53 | *Lysinibacillus macroides* | 1580 | 1580 | 100% | 99.42% |
| C3-56 | *Bacillus wiedmannii/proteolyticus/fungorum* | 1644 | 1644 | 100% | 100.00% |
| C3-59 | *Bacillus subtilis* | 1592 | 1592 | 100% | 100.00% |
| C3-61 | *Bacillus mobilis* | 1622 | 1622 | 100% | 100.00% |
| C3-62 | *Bacillus subtilis* | 1592 | 1592 | 100% | 100.00% |
| C3-63 | *Lysinibacillus pakistanensis* | 1609 | 1609 | 100% | 100.00% |
| C3-66 | *Bacillus pumilus/zhangzhouensis/safensis* | 1602 | 1602 | 100% | 100.00% |
| C3-67 | *Lysinibacillus macroides* | 1580 | 1580 | 100% | 99.42% |
| C3-70 | *Lysinibacillus macroides* | 1580 | 1580 | 100% | 99.42% |
| C3-72 | *Bacillus subtilis* | 1591 | 1591 | 100% | 100.00% |
| C3-73 | *Lysinibacillus pakistanensis* | 1613 | 1613 | 100% | 100.00% |
| C3-76 | *Lysinibacillus pakistanensis* | 1613 | 1613 | 100% | 100.00% |

**Table S4**. Initial evaluation of extracellular enzyme production and various PGP characteristics for all isolates.

| **Species** | **Isolate** | **Growth +N_2_** | **Growth -N_2_** | **Sol P** | **EPS** | **Swarm** | **Swim** | **Amy** | **Prot** | **Xyl** | **Mann** | **Cell** | **Gel** | **Pec** |
| --- | --- | --- | --- | --- | --- | --- | --- | --- | --- | --- | --- | --- | --- | --- |
|  |  |  |  |  |  |  |  | EI | EI | EI | EI | EI | EI | EI |
| *Lysinibacillus macroides* | ED1-1 | + + | + + | ─ | ─ | ─ | + + + | ─ | ─ | ─ | ─ | ─ | ─ | ─ |
| *Cytobacillus ciccensis* | ED1-2 | + + | + | ─ | ─ | ─ | + + + | ─ | ─ | ─ | ─ | ─ | ─ | ─ |
| *Bacillus badius* | ED1-3 | + + | + | ─ | ─ | ─ | + + + | ─ | ─ | ─ | ─ | ─ | ─ | ─ |
| *Lysinibacillus fusiformis* | ED1-4 | + + | + | ─ | ─ | + | + + + | ─ | ─ | ─ | ─ | ─ | ─ | ─ |
| *Paenibacillus taichungensis* | ED2-1 | + + | + | ─ | ─ | ─ | + + + | 2.5 | ─ | 1.1 | ─ | 1.3 | 1.1 | 1.3 |
| *Bacillus velezensis* | ED2-2 | + + | + | ─ | ─ | + + + | + + + | 1.3 | 1.3 | ─ | 1.7 | 1.2 | 1.9 | 1.5 |
| *Knoellia locipacati* | ED2-4 | + | ─ | ─ | ─ | ─ | ─ | ─ | ─ | ─ | ─ | ─ | ─ | ─ |
| *Curtobacterium pusillum* | ED2-6 | + | + | + | ─ | + | + + | ─ | ─ | ─ | ─ | ─ | 1.8 | ─ |
| *Curtobacterium pusillum* | ED2-7 | + | + + | + | ─ | ─ | ─ | ─ | ─ | ─ | ─ | ─ | 1.8 | ─ |
| *Microbacterium thalassium* | ED2-8 | + + | + | ─ | ─ | ─ | ─ | 1.4 | ─ | ─ | ─ | ─ | ─ | ─ |
| *Mycetocola manganoxydans* | ED2-10 | + | ─ | ─ | ─ | ─ | ─ | 1.3 | ─ | ─ | ─ | ─ | ─ | ─ |
| *Staphylococcus succinus* subsp*. succinus* | ED3-1 | + + | + | + | ─ | ─ | + + + | ─ | ─ | ─ | ─ | ─ | 1.6 | ─ |
| *Pseudomonas psychrotolerans* | ED3-2 | + + + | + + | + + | + | + + + | + | ─ | ─ | ─ | ─ | ─ | ─ | ─ |
| *Bacillus mobilis* | ED3-3 | + | + | ─ | ─ | + + + | + | 1.7 | ─ | ─ | ─ | 1.1 | 1.9 | ─ |
| *Kosakonia cowanii* | ED3-4 | + + | + | + + | + + + ^d^ | + + | + + + | ─ | ─ | ─ | ─ | ─ | ─ | ─ |
| *Providencia vermicola* | ED3-10 | + + | + | + | ─ | + | + + + | ─ | ─ | ─ | ─ | ─ | ─ | ─ |
| *Acinetobacter lactucae* | ED3-13 | + + | + | + + | ─ | ─ | ─ | ─ | ─ | ─ | ─ | ─ | ─ | ─ |
| *Pseudomonas psychrotolerans* | ED3-21 | + + | + | + + | ─ | ─ | + + | ─ | ─ | ─ | ─ | ─ | ─ | ─ |
| *Pseudomonas psychrotolerans* | ED3-42 | + | + | + + | ─ | ─ | ─ | ─ | ─ | ─ | ─ | ─ | ─ | ─ |
| *Microbacterium testaceum* | ED3-43 | + | + | ─ | ─ | ─ | ─ | ─ | ─ | ─ | ─ | ─ | ─ | ─ |
| *Acinetobacter lactucae* | ED3-46 | + + | + | + + | ─ | ─ | ─ | ─ | ─ | ─ | ─ | ─ | ─ | ─ |
| *Providencia sneebia* | ED3-48 | + + | + | + + | + | + | ─ | ─ | ─ | ─ | ─ | ─ | ─ | ─ |
| *Klebsiella michiganensis* | ED3-48/1 | + + | + | + + | + | ─ | ─ | ─ | ─ | ─ | ─ | ─ | ─ | ─ |
| *Bacillus zhangzhouensis* | ED3-74 | + + | + | ─ | ─ | + + + | + + + | 1.2 | ─ | ─ | 1.6 | 1.6 | 1.9 | ─ |
| *Erwinia persicina* | ED3-74/1 | + + | + | + + | + + | + + + | + + + | ─ | ─ | ─ | ─ | ─ | ─ | ─ |
| *Glutamicibacter mishrai* | ED3-75 | + + | + | ─ | ─ | ─ | ─ | 1.8 | 2.0 | ─ | 1.2 | ─ | 1.9 | ─ |
| *Sanguibacter keddieii* | ED3-76 | + + | + | ─ | ─ | ─ | ─ | 1.5 | ─ | ─ | 1.3 | ─ | 1.9 | ─ |
| *Micrococcus luteus* | ED3-77 | + | + + | ─ | ─ | ─ | ─ | ─ | ─ | ─ | ─ | ─ | 1.1 | ─ |
| *Erwinia tasmaniensis* | ED3-78 | + | + + | + | + + + ^d^ | + + + | + + + | ─ | ─ | ─ | ─ | ─ | ─ | ─ |
| *Erwinia tasmaniensis* | ED3-79 | + | + + | + | + + + ^d^ | + + + | + + + | ─ | ─ | ─ | ─ | ─ | ─ | ─ |
| *Curtobacterium pusillum* | ED3-81 | + | + | ─ | ─ | ─ | + | ─ | ─ | ─ | ─ | ─ | 1.2 | ─ |
| *Erwinia persicina* | ED3-82 | + + | + | + + + | + + + | + + + | + + + | ─ | ─ | ─ | ─ | ─ | ─ | ─ |
| *Micrococcus aloeverae* | ED3-83 | + | + | ─ | ─ | ─ | ─ | ─ | ─ | ─ | 1.4 | ─ | ─ | ─ |
| *Pseudomonas psychrotolerans* | ED3-84 | + + | + | + + | ─ | ─ | + + + | ─ | ─ | ─ | ─ | ─ | ─ | ─ |
| *Alkalihalobacillus rhizosphaerae* | ED3-85 | + + | + | ─ | ─ | ─ | ─ | ─ | ─ | ─ | ─ | ─ | ─ | ─ |
| *Frigoribacterium faeni* | ED3-86 | + | ─ | ─ | ─ | ─ | ─ | ─ | ─ | ─ | ─ | 1.1 | ─ | ─ |
| *Bacillus subtilis* | ED3-89 | + | + + | + | ─ | + + + | + + + | ─ | ─ | ─ | 1.1 | 1.6 | 1.5 | 1.2 |
| *Lysinibacillus macroides* | T1-1 | + | + | ─ | ─ | ─ | + + + | ─ | ─ | ─ | ─ | ─ | ─ | ─ |
| *Priestia aryabhattai* | T1-2 | + + | + | + | + | + | ─ | ─ | ─ | ─ | ─ | ─ | 1.9 | ─ |
| *Bacillus wiedmannii/proteolyticus/fungorum* | T1-3 | + + | + + | ─ | + | ─ | + + + | ─ | ─ | ─ | ─ | ─ | 2.3 | ─ |
| *Lysinibacillus macroides* | T1-4 | + + | + + | ─ | ─ | ─ | + + + | ─ | ─ | ─ | ─ | ─ | ─ | ─ |
| *Bacillus altitudinis/aerophilus/stratosphericus* | T1-5 | + + | + + | ─ | + | + + + | + + + | ─ | ─ | ─ | ─ | ─ | 1.1 | ─ |
| *Bacillus altitudinis/aerophilus/stratosphericus* | T1-6 | + + | + | + | ─ | ─ | + + + | ─ | ─ | ─ | ─ | ─ | 1.6 | ─ |
| *Lysinibacillus pakistanensis* | T1-6/1 | + + | + | ─ | ─ | ─ | + + + | ─ | ─ | ─ | ─ | ─ | ─ | ─ |
| *Bacillus halotolerans* | T2-1 | + + | + | + | ─ | + + + | + + + | 1.7 | 1.2 | ─ | 1.8 | 2.2 | 1.8 | ─ |
| *Paenibacillus azotifigens* | T2-2 | + + | + | ─ | ─ | ─ | ─ | 1.6 | ─ | ─ | ─ | 1.7 | ─ | ─ |
| *Neobacillus niacini* | T2-6 | + | + | ─ | ─ | ─ | ─ | ─ | ─ | ─ | ─ | ─ | ─ | ─ |
| *Neorhizobium huautlense* | T2-8 | + | + | ─ | + | ─ | + | ─ | ─ | ─ | ─ | 1.2 | ─ | ─ |
| *Micrococcus aloeverae* | T2-9 | + + | + + | ─ | ─ | ─ | ─ | ─ | ─ | ─ | 1.4 | ─ | ─ | ─ |
| *Kocuria rosea* | T2-14 | + + | + | ─ | ─ | ─ | ─ | 1.4 | ─ | ─ | ─ | ─ | ─ | ─ |
| *Paenibacillus catalpae/lupini* | T2-15 | + + | + | ─ | ─ | ─ | + | 1.3 | ─ | 1.2 | 1.4 | ─ | ─ | ─ |
| *Rhodococcus corynebacterioides* | T2-16 | + | + | ─ | + | ─ | ─ | ─ | ─ | ─ | ─ | ─ | ─ | ─ |
| *Rathayibacter tritici* | T2-18 | + | + | ─ | ─ | ─ | ─ | ─ | ─ | ─ | ─ | ─ | ─ | ─ |
| *Microbacterium testaceum* | T2-19 | + | + | ─ | + | ─ | ─ | ─ | ─ | ─ | ─ | ─ | 1.3 | ─ |
| *Paenibacillus lautus* | T2-20 | + | ─ | ─ | ─ | ─ | + | 1.3 | ─ | ─ | ─ | ─ | ─ | ─ |
| *Rhodococcus cerastii* | T2-21 | + + | + | + | + | + | ─ | ─ | ─ | ─ | ─ | ─ | ─ | ─ |
| *Micrococcus luteus* | T2-22 | + + | + | ─ | ─ | + | + | ─ | ─ | ─ | ─ | ─ | 1.1 | ─ |
| *Bacillus velezensis* | T2-23 | + + | + | ─ | ─ | + + + | + + + | 1.4 | 1.2 | ─ | 1.8 | 1.5 | 1.7 | 1.7 |
| *Arthrobacter oryzae* | T2-25 | + + | + | ─ | ─ | ─ | ─ | 1.3 | ─ | 1.5 | 1.4 | ─ | 1.7 | ─ |
| *Micrococcus aloeverae* | T2-26 | + + | + | ─ | ─ | + | + | ─ | ─ | ─ | ─ | ─ | 1.7 | ─ |
| *Metabacillus indicus* | T2-28 | + + | + + | ─ | ─ | ─ | + | 1.3 | ─ | ─ | ─ | ─ | ─ | ─ |
| *Massilia timonae* | T2-32 | + | + + | ─ | ─ | ─ | + + + | 2.4 | 1.1 | 1.9 | 1.5 | ─ | 1.8 | ─ |
| *Bacillus sonorensis* | T3-3 | + + | + | + | ─ | ─ | + + + | 1.3 | ─ | ─ | 1.4 | ─ | ─ | ─ |
| *Bacillus subtilis* | T3-4 | + + | + | + | ─ | + + + | + + + | 1.3 | 1.2 | ─ | 1.8 | 1.8 | 1.5 | 2.0 |
| *Bacillus sonorensis* | T3-5 | + + | + | + | ─ | + + + | + + + | 1.3 | ─ | 1.3 | 1.4 | 2.0 | 1.2 | ─ |
| *Bacillus sonorensis* | T3-6 | + + | + | + | ─ | + + + | + + + | 1.2 | ─ | ─ | 1.7 | 1.9 | 1.3 | ─ |
| *Acinetobacter lactucae* | T3-6/1 | + + | + | + + | ─ | ─ | ─ | ─ | ─ | ─ | ─ | ─ | ─ | ─ |
| *Lysinibacillus pakistanensis* | TF1-1 | + | + | ─ | ─ | + | + + + | ─ | ─ | ─ | ─ | ─ | ─ | ─ |
| *Bacillus mobilis* | TF1-2 | + + | ─ | ─ | + | + | + + + | 1.6 | ─ | ─ | ─ | ─ | ─ | ─ |
| *Lysinibacillus fusiformis* | TF1-3 | + + | + | ─ | ─ | ─ | + + + | ─ | 1.5 | ─ | ─ | ─ | ─ | ─ |
| *Lysinibacillus macroides* | TF1-4 | + + | + | ─ | ─ | ─ | + + + | ─ | ─ | ─ | ─ | ─ | ─ | ─ |
| *Bacillus subtilis* | TF2-1 | + + | + + | + | + | + + + | + + + | 1.1 | 1.1 | ─ | ─ | ─ | 1.6 | ─ |
| *Micrococcus luteus/aloeverae* | TF2-6 | + | + | ─ | ─ | ─ | ─ | ─ | ─ | ─ | ─ | ─ | ─ | ─ |
| *Bacillus subtilis* | TF3-6 | + | + + | + | + | + + + | + + + | 1.7 | 1.1 | 2.3 | ─ | ─ | 1.5 | 2.3 |
| *Bacillus subtilis* | TF3-7/1 | + + | + | + | + | + + + | + + + | 1.1 | ─ | 2.0 | 1.4 | ─ | 1.8 | 1.9 |
| *Bacillus licheniformis* | TF3-16 | + + | + + | + | ─ | + + | + + + | 1.4 | ─ | ─ | 1.2 | ─ | 1.3 | ─ |
| *Microbacterium paludicola* | TF3-20 | + | + | ─ | ─ | ─ | + | ─ | ─ | ─ | ─ | ─ | 1.2 | ─ |
| *Paenibacillus lautus* | TF3-27 | + + | ─ | ─ | ─ | ─ | + + + | 1.3 | ─ | ─ | ─ | ─ | ─ | ─ |
| *Weizmannia ginsengihumi* | TF3-29 | + | + | + | + | ─ | ─ | ─ | ─ | ─ | ─ | ─ | ─ | ─ |
| *Bacillus subtilis* | TF3-32 | + + + | + + | ─ | ─ | + + + | + + + | 1.4 | 1.1 | ─ | ─ | 2.2 | 1.9 | ─ |
| *Brevundimonas nasdae/vesicularis* | TF3-33 | ─ | ─ | ─ | ─ | ─ | + | ─ | ─ | ─ | ─ | ─ | ─ | ─ |
| *Bacillus oleivorans* | TF3-82 | + | + | ─ | ─ | ─ | ─ | ─ | ─ | ─ | ─ | ─ | ─ | ─ |
| *Bacillus pacificus/paranthracis* | KO1-1 | + | + | + | ─ | + | + + + | 1.6 | ─ | ─ | ─ | 1.3 | 2.1 | ─ |
| *Rossellomorea marisflavi* | KO1-2 | + | + | ─ | ─ | + | + + + | ─ | ─ | ─ | ─ | ─ | 1.7 | ─ |
| *Lysinibacillus macroides* | KO1-3 | + | + | ─ | ─ | ─ | + + + | ─ | ─ | ─ | ─ | ─ | ─ | ─ |
| *Rossellomorea marisflavi* | KO1-3/2 | + | ─ | ─ | ─ | ─ | + + + | ─ | ─ | ─ | 1.1 | ─ | 1.8 | ─ |
| *Bacillus wiedmannii/proteolyticus/fungorum* | KO1-4 | + | + | ─ | ─ | + + | ─ | 1.2 | ─ | ─ | 1.1 | ─ | 1.8 | ─ |
| *Lysinibacillus macroides* | KO1-5 | + | + | ─ | ─ | ─ | + + + | 1.1 | ─ | ─ | ─ | ─ | ─ | ─ |
| *Brevibacillus reuszeri* | KO1-7 | + | ─ | ─ | ─ | ─ | + + + | ─ | ─ | ─ | ─ | ─ | ─ | ─ |
| *Micrococcus aloeverae* | KO2-1 | + | + | ─ | ─ | ─ | ─ | ─ | ─ | ─ | ─ | ─ | ─ | ─ |
| *Micrococcus aloeverae* | KO2-2 | + | + | ─ | ─ | + + | + + + | ─ | ─ | ─ | ─ | ─ | 1.2 | ─ |
| *Micrococcus terreus* | KO2-3 | + + | + + | ─ | ─ | ─ | ─ | ─ | ─ | ─ | ─ | ─ | 1.2 | ─ |
| *Micrococcus aloeverae* | KO2-4 | + | + | ─ | ─ | ─ | ─ | ─ | 2.0 | ─ | 1.5 | ─ | 1.6 | ─ |
| *Staphylococcus epidermidis* | KO2-6 | + + | ─ | + | ─ | ─ | ─ | ─ | ─ | ─ | ─ | ─ | ─ | ─ |
| *Kosakonia cowanii* | KO3-2 | + | + | + + | + + + ^d^ | + + | + + + | ─ | ─ | ─ | ─ | ─ | ─ | ─ |
| *Bacillus haynesii* | KO3-3 | + + | + | ─ | ─ | + + + | + + + | ─ | ─ | ─ | 1.4 | 1.6 | ─ | 1.0 |
| *Micrococcus aloeverae* | KO3-4 | + | + | ─ | ─ | + | ─ | ─ | ─ | ─ | ─ | ─ | ─ | ─ |
| *Mixta theicola* | KO3-9 | + + | + + + | + | + + + | + + + | + | ─ | ─ | ─ | ─ | ─ | ─ | ─ |
| *Alkalihalobacillus gibsonii* | KO3-10 | + | ─ | + | ─ | + + | + | ─ | ─ | ─ | ─ | ─ | ─ | 1.5 |
| *Bacillus subtilis* | KO3-11/1 | + + | + + | + | + | + + + | + | 1.2 | ─ | ─ | ─ | 1.2 | 1.3 | 2.4 |
| *Bacillus zhangzhouensis* | KO3-12 | + | + | ─ | ─ | + | + | ─ | 1.1 | ─ | ─ | ─ | 1.8 | 1.2 |
| *Enterococcus gallinarum* | KO3-14 | + | ─ | + | + + + ^d^ | + | + + | ─ | ─ | ─ | ─ | ─ | ─ | ─ |
| *Pseudomonas psychrotolerans* | KO3-17 | + + | + | + + | ─ | ─ | + + + | ─ | ─ | ─ | ─ | ─ | ─ | ─ |
| *Bacillus subtilis* | KO3-18 | + | + + | + | + | + + + | + + + | 1.1 | ─ | ─ | ─ | 1.8 | 1.4 | 2.4 |
| *Microbacterium saccharophilum* | KO3-19 | + + | + + | + + | + | + + + | + + + | ─ | ─ | ─ | ─ | ─ | ─ | ─ |
| *Curtobacterium pusillum* | KO3-20 | + + | + + | ─ | + | ─ | + | ─ | ─ | ─ | ─ | ─ | 1.9 | ─ |
| *Bacillus subtilis* | KO3-26 | + + + | + | + | ─ | + + + | + + + | 1.6 | ─ | ─ | ─ | 1.1 | 1.7 | 2.1 |
| *Pantoea agglomerans/Curtobacterium plantarum* | KO3-28 | + | + + | + + | + + + | + | + + | ─ | ─ | ─ | ─ | ─ | ─ | ─ |
| *Pantoea agglomerans* | KO3-28/1 | + | + | + + | + + + | + | + + | ─ | ─ | ─ | ─ | ─ | ─ | ─ |
| *Pantoea allii* | KO3-30 | + | + | + | + + + | + | ─ | ─ | ─ | ─ | ─ | ─ | ─ | ─ |
| *Pseudomonas psychrotolerans* | KO3-32 | + + | + | + | + | ─ | ─ | ─ | ─ | ─ | ─ | ─ | ─ | 1.1 |
| *Okibacterium fritillariae* | KO3-36 | + | ─ | ─ | ─ | ─ | ─ | ─ | ─ | ─ | ─ | ─ | ─ | ─ |
| *Bacillus haynesii* | KO3-38 | + + | + | + | ─ | + + + | + + + | 1.4 | 1.1 | ─ | ─ | ─ | 1.3 | ─ |
| *Sanguibacter inulinus* | KO3-39 | + | ─ | ─ | ─ | ─ | + + | ─ | ─ | ─ | ─ | ─ | ─ | ─ |
| *Mixta theicola* | KO3-44 | + + | + + + | + | + + + ^d^ | + | + + + | ─ | ─ | ─ | ─ | ─ | ─ | ─ |
| *Microbacterium arborescens/imperiale* | KO3-46 | + + | + | ─ | ─ | ─ | ─ | ─ | ─ | ─ | ─ | ─ | ─ | ─ |
| *Curtobacterium pusillum* | KO3-47 | + | + | ─ | + | ─ | ─ | ─ | ─ | ─ | ─ | ─ | 1.8 | ─ |
| *Bacillus zhangzhouensis* | KO3-48 | + + | + | ─ | ─ | + + + | + + + | ─ | ─ | ─ | ─ | ─ | 1.7 | 1.2 |
| *Bacillus zhangzhouensis* | KO3-48/1 | + + | + | ─ | ─ | + + + | + + + | ─ | ─ | ─ | ─ | ─ | 1.7 | 1.2 |
| *Microbacterium saccharophilum* | KO3-49 | + + | + | ─ | ─ | ─ | ─ | ─ | ─ | ─ | ─ | ─ | ─ | ─ |
| *Curtobacterium pusillum* | KO3-51 | + + | + + | ─ | + | ─ | + | ─ | ─ | ─ | ─ | ─ | 1.9 | ─ |
| *Curtobacterium pusillum* | KO3-80 | + + | + + | ─ | + | ─ | + | ─ | ─ | ─ | ─ | ─ | 1.9 | ─ |
| *Bacillus toyonensis/thuringiensis* | C1-1 | + | + | ─ | ─ | ─ | + + + | 1.8 | 1.1 | ─ | ─ | ─ | 1.7 | ─ |
| *Bacillus pacificus/paranthracis* | C1-2 | + | + | ─ | + | ─ | + + + | ─ | ─ | ─ | ─ | ─ | 1.7 | ─ |
| *Lysinibacillus macroides* | C1-3 | + | + | ─ | ─ | ─ | + + + | ─ | ─ | ─ | ─ | ─ | ─ | ─ |
| *Brevibacillus borstelensis* | C1-5 | + + | ─ | ─ | ─ | ─ | ─ | ─ | ─ | ─ | ─ | ─ | ─ | ─ |
| *Bacillus pseudomycoides* | C2-1 | + | ─ | ─ | ─ | + | + + + | 2.8 | 1.0 | ─ | ─ | ─ | ─ | ─ |
| *Micrococcus luteus* | C2-5 | + + | + | ─ | ─ | ─ | ─ | ─ | ─ | ─ | ─ | ─ | ─ | ─ |
| *Micrococcus luteus* | C2-6 | + | + + | ─ | ─ | ─ | ─ | ─ | ─ | ─ | ─ | ─ | ─ | ─ |
| *Massilia timonae* | C2-7 | + | + | ─ | ─ | ─ | + + | 2.9 | ─ | ─ | ─ | ─ | 1.4 | ─ |
| *Massilia timonae* | C2-7/1 | + + | + + | ─ | ─ | ─ | + + + | 2.8 | ─ | ─ | ─ | ─ | 1.4 | ─ |
| *Corynebacterium doosanense* | C3-13 | + | + | ─ | ─ | + + | ─ | ─ | ─ | ─ | ─ | ─ | ─ | ─ |
| *Bacillus mojavensis* | C3-16/2 | + + | + | + | + | + + + | + + + | ─ | ─ | ─ | ─ | 1.2 | ─ | 2.5 |
| *Bacillus halotolerans* | C3-16/2.1 | + + | + + | + | ─ | + + + | + + + | 1.2 | 1.8 | ─ | 1.5 | 1.8 | 1.3 | 2.0 |
| *Bacillus halotolerans* | C3-16/2.3 | + | + + | + | ─ | + + + | + + + | 1.3 | 1.9 | ─ | 1.6 | 1.7 | 1.8 | 1.9 |
| *Curtobacterium pusillum* | C3-17 | + | + | ─ | + | ─ | ─ | ─ | ─ | ─ | ─ | ─ | ─ | ─ |
| *Pseudoclavibacter helvolus* | C3-18 | + | + | ─ | ─ | ─ | ─ | ─ | ─ | ─ | ─ | ─ | ─ | ─ |
| *Bacillus amyloliquefaciens* | C3-19 | + + + | + | + | ─ | + + + | + + + | ─ | 1.2 | ─ | 1.7 | 1.6 | 1.6 | 1.6 |
| *Paenibacillus polymyxa* | C3-36 | + + | + + | ─ | + | ─ | + + | 1.6 | ─ | 1.6 | 1.2 | 2.0 | 1.4 | 1.3 |
| *Bacillus subtilis* | C3-40 | + | + | + | ─ | ─ | + + + | ─ | ─ | ─ | ─ | ─ | 1.6 | ─ |
| *Pantoea agglomerans/Curtobacterium plantarum* | C3-44 | + + + | + + + | + | + + + | ─ | + + + | ─ | ─ | ─ | ─ | ─ | ─ | ─ |
| *Bacillus altitudinis/aerophilus/stratosphericus* | C3-47 | + + | + + | + | + | + + + | + + + | ─ | 1.1 | ─ | ─ | ─ | 1.8 | ─ |
| *Bacillus zhangzhouensis* | C3-50 | + | + | + | + + | + | + + + | ─ | 1.2 | ─ | ─ | ─ | 1.7 | ─ |
| *Bacillus zhangzhouensis* | C3-50/1 | + | + | ─ | ─ | + + + | + + + | ─ | 1.1 | 1.2 | ─ | ─ | 1.9 | ─ |
| *Lysinibacillus macroides* | C3-53 | + + | + | + | ─ | + + + | + + + | ─ | ─ | ─ | ─ | ─ | ─ | ─ |
| *Bacillus wiedmannii/proteolyticus/fungorum* | C3-56 | + + | + | ─ | + | ─ | + + + | 1.5 | ─ | ─ | ─ | ─ | 2.2 | ─ |
| *Bacillus subtilis* | C3-59 | + + | + + | + | ─ | + + | + + + | 1.4 | 1.1 | ─ | ─ | ─ | 1.4 | ─ |
| *Bacillus mobilis* | C3-61 | + | ─ | ─ | ─ | ─ | + + + | 1.7 | 1.4 | ─ | ─ | ─ | 1.7 | ─ |
| *Bacillus subtilis* | C3-62 | + + | + | ─ | + | ─ | + + + | ─ | ─ | ─ | 1.4 | ─ | 1.5 | 1.8 |
| *Lysinibacillus pakistanensis* | C3-63 | + | + | ─ | ─ | + + + | + + + | ─ | ─ | ─ | ─ | ─ | ─ | ─ |
| *Bacillus pumilus/zhangzhouensis/safensis* | C3-66 | + + | ─ | ─ | + + | + + + | + + + | ─ | ─ | ─ | ─ | ─ | 1.5 | ─ |
| *Lysinibacillus macroides* | C3-67 | + | ─ | ─ | ─ | + + | ─ | ─ | 1.3 | ─ | ─ | ─ | ─ | ─ |
| *Lysinibacillus macroides* | C3-70 | + | ─ | ─ | ─ | ─ | + + + | 1.6 | 1.5 | ─ | ─ | ─ | 1.3 | ─ |
| *Bacillus subtilis* | C3-72 | + + | + | + | + | + + + | + + + | 1.5 | 1.6 | ─ | ─ | 1.7 | 1.5 | ─ |
| *Lysinibacillus pakistanensis* | C3-73 | + | ─ | ─ | ─ | ─ | + + + | ─ | ─ | ─ | ─ | ─ | ─ | ─ |
| *Lysinibacillus pakistanensis* | C3-76 | + | + | ─ | ─ | + | + + + | ─ | ─ | ─ | ─ | ─ | ─ | ─ |

─ no activity; + weak activity; ++ good activity; +++ very good activity; +++^d^ excellent activity. EI - Enzymatic index (EI >1 good activity; EI=1-2 very good activity; EI>2 excellent activity). Sol P - Phosphate solubilization; EPS - Exopolysaccharides production; Swarm - Swarming motility; Swim - Swimming motility; Amy - Amylases; Prot - Proteases; Xyl - Xylanases; Mann - Mannanases; Cell - Cellulases; Gel - Gelatinases;	Pec - Pectinases. Eduarda (ED), Koala (KO), Tibor (T), Tajfun (TF) and *Cercospora*-resistant (C) - numbers beside hybrid abbreviations indicate the origin of the isolate i.e. rhizosphere (1), root (2), seed (3).

**Table S5**. Evaluation of growth at different concentrations of NaCl and PEG for selected strains.

| **Species** | **Isolates** | **Origin** | **NaCl** | | | | | **PEG** | | | |
| --- | --- | --- | --- | --- | --- | --- | --- | --- | --- | --- | --- |
|  |  |  | **1%** | **3%** | **5%** | **8%** | **10%** | **5%** | **10%** | **20%** | **30%** |
| *Bacillus halotolerans* | C3-16/2.1 | seed | + + + | + + + | + + + | + + | + + | + + + | + + + | + + | + + |
| *Bacillus amyloliquefaciens* | C3-19 | seed | + + + | + + + | + + | + | ─ | + + + | + + + | + + + | + + |
| *Paenibacillus polymyxa* | C3-36 | seed | + | ─ | ─ | ─ | ─ | + + + | + + + | ─ | ─ |
| *Bacillus zhangzhouensis* | C3-50 | seed | + + + | + + + | + + + | + + | ─ | + + + | + + + | + + + | + + + |
| *Lysinibacillus macroides* | C3-53 | seed | + + + | + + | + | ─ | ─ | + + + | + + + | + + | + + |
| *Bacillus subtilis* | C3-59 | seed | + + + | + + | + + | + | + | + + | + + + | + + | + |
| *Bacillus subtilis* | C3-62 | seed | + + + | + + | + + | + | ─ | + + + | + + + | + + + | + + + |
| *Bacillus subtilis* | C3-72 | seed | + + + | + + | + + | + + | + | + + + | + + + | + + | + + |
| *Paenibacillus taichungensis* | ED2-1 | root | + + + | + + | ─ | ─ | ─ | + + | + + | + | ─ |
| *Bacillus velezensis* | ED2-2 | root | + + + | + + + | + + | + | + | + + + | + + + | + + + | + + |
| *Curtobacterium pusillum* | ED2-6 | root | + + + | + + | ─ | ─ | ─ | + + + | + + | + + | + |
| *Providencia vermicola* | ED3-10 | seed | + + + | + + + | + + + | + | ─ | + + + | + + + | + + | + |
| *Glutamicibacter mishrai* | ED3-75 | seed | + + + | + + | + + | + + | + | + + + | + + | + + | + |
| *Erwinia tasmaniensis* | ED3-79 | seed | + + + | + + + | + + | ─ | ─ | + + + | + + + | + + | + |
| *Bacillus subtilis* | ED3-89 | seed | + + + | + + + | + + | + + | + | + + + | + + | + + | + + |
| *Bacillus pacificus/paranthracis* | KO1-1 | rhiz. | + + + | + + | + | ─ | ─ | + + + | + + | ─ | ─ |
| *Bacillus subtilis* | KO3-11/1 | seed | + + + | + + | + + | + | ─ | + | + + | + | + |
| *Bacillus subtilis* | KO3-18 | seed | + + + | + + | + + | + + | + | + + + | + + + | + + | + + |
| *Pseudomonas oryzihabitans* | KO3-19 | seed | + + + | + + | + | ─ | ─ | + + + | + + | + + | + + |
| *Bacillus subtilis* | KO3-26 | seed | + + + | + + + | + + | + | ─ | + + + | + + + | + + | + + |
| *Mixta theicola* | KO3-44 | seed | + + + | + + + | + + | + | ─ | + + + | + + + | + + | + |
| *Priestia aryabhattaii* | T1-2 | rhiz. | + + + | + + + | + + | + | ─ | + + + | + + | + + | + |
| *Bacillus halotolerans* | T2-1 | root | + + + | + + + | + + | + + | ─ | + + | + + | + + | + + + |
| *Bacillus velezensis* | T2-23 | root | + + + | + + + | + + | + + | + | + + + | + + + | + + + | + + |
| *Arthrobacter oryzae* | T2-25 | root | + + + | + + | ─ | ─ | ─ | + + + | + + + | + + | + |
| *Micrococcus aloeverae* | T2-26 | root | + + + | + + | + | + | + | + + + | + + + | + + | + + |
| *Bacillus subtilis* | T3-4 | seed | + + + | + + | + | + | ─ | + + + | + + + | + + | + + |
| *Bacillus sonorensis* | T3-5 | seed | + + + | + + + | + + | + | ─ | + + | + + + | + + | + + |
| *Bacillus subtilis* | TF2-1 | root | + + + | + + + | + + | + + | + | + + + | + + + | + + + | + + + |
| *Bacillus subtilis* | TF3-6 | seed | + + + | + + + | + + | + + | + | + + + | + + + | + + | + + |
| *Bacillus subtilis* | TF3-7/1 | seed | + + + | + + | + + | + + | + | + + + | + + | + | + + |
| *Bacillus subtilis* | TF3-32 | seed | + + + | + + + | + + | + + | + | + + | + + | + + | + |

─ no growth; + weak growth, + + good growth; + + + excellent growth

**Table S6.** Read retention in five different sugar beet seed samples after each of the processing phases.

| **Sample** |  | **Input** | **Adapter removal** | **Filtered** | **Denoised_F** | **Denoised_R** | **Merged** | **Chimera removal** | **Chloroplast +mitochondria + undefined kingdom removal** | **Final %** |
| --- | --- | --- | --- | --- | --- | --- | --- | --- | --- | --- |
| TF1.1 |  | 105248 | 104264 | 62487 | 61950 | 61803 | 61158 | 58541 | 3658 | 3,48% |
| TF1.2 |  | 111036 | 110562 | 87648 | 87260 | 87339 | 86680 | 83247 | 3946 | 3,55% |
| TF1.3 |  | 116831 | 116194 | 87151 | 86811 | 86914 | 86456 | 83274 | 1285 | 1,10% |
| ED1.1 |  | 116927 | 115977 | 85270 | 84287 | 83985 | 82014 | 76499 | 6231 | 5,33% |
| ED1.2 |  | 104409 | 103697 | 79438 | 78249 | 77888 | 75313 | 69646 | 7486 | 7,17% |
| ED1.3 |  | 109353 | 108312 | 76639 | 75469 | 75179 | 73213 | 68998 | 5833 | 5,33% |
| KO1.1 |  | 116652 | 115753 | 79789 | 78885 | 78613 | 77236 | 73485 | 3606 | 3,09% |
| KO1.2 |  | 103073 | 102414 | 81388 | 80415 | 80131 | 78430 | 72304 | 6920 | 6,71% |
| KO1.3 |  | 118360 | 117804 | 95333 | 94478 | 94206 | 92234 | 86158 | 4235 | 3,58% |
| T1.1 |  | 102613 | 101721 | 72203 | 71387 | 71221 | 69809 | 66016 | 4524 | 4,41% |
| T1.2 |  | 110570 | 109453 | 85930 | 84806 | 84293 | 82111 | 76010 | 8216 | 7,43% |
| T1.3 |  | 114251 | 113606 | 88201 | 86828 | 86106 | 83499 | 78459 | 5085 | 4,45% |
| C1.1 |  | 101470 | 100661 | 68275 | 67433 | 67081 | 65743 | 62361 | 4014 | 3,96% |
| C1.2 |  | 102891 | 102383 | 82522 | 82214 | 82184 | 81711 | 77900 | 2982 | 2,90% |
| C1.3 |  | 117585 | 116969 | 93107 | 92702 | 92786 | 92232 | 87254 | 2288 | 1,95% |

Eduarda (ED), Koala (KO), Tibor (T), Tajfun (TF) and *Cercospora*-resistant (C)
